# ﻿Hidden in the riffles: A new suckermouth catfish (Mochokidae, *Chiloglanis*) from the middle Zambezi River system, Zimbabwe

**DOI:** 10.3897/zookeys.1197.114679

**Published:** 2024-04-04

**Authors:** Tadiwa I. Mutizwa, Wilbert T. Kadye, Pedro H. N. Bragança, Taurai Bere, Albert Chakona

**Affiliations:** 1 Department of Ichthyology and Fisheries Science, Faculty of Science, Rhodes University, Prince Alfred Street, PO Box 94, Makhanda, 6140, South Africa NRF-South African Institute for Aquatic Biodiversity Makhanda South Africa; 2 NRF-South African Institute for Aquatic Biodiversity, Somerset Street, Private Bag 1015, Makhanda, 6140, South Africa Rhodes University Makhanda South Africa; 3 Department of Ichthyology, American Museum of Natural History, Central Park West at 79th Street, New York, NY 10024, USA Department of Ichthyology, American Museum of Natural History New York United States of America; 4 School of Wildlife, Ecology and Conservation, Chinhoyi University of Technology, Private Bag 7724, Chinhoyi, Zimbabwe Chinhoyi University of Technology Chinhoyi Zimbabwe

**Keywords:** Diversity, freshwater, integrative taxonomy, rheophilic taxa, southern Africa

## Abstract

The recent surge in the discovery of hidden diversity within rheophilic taxa, particularly in West and East Africa, prompted a closer examination of the extent to which the current taxonomy may obscure the diversity of riffle-dwelling suckermouth catfishes in the genus *Chiloglanis* in southern Africa. Currently, the region comprises eight valid species within this genus. Seven of them have relatively narrow geographic distribution ranges except for *C.neumanni*, which is considered to be widely distributed, occurring from the Buzi River system in the south, and its northern limit being the eastward draining river systems in Tanzania. Recent surveys of the middle Zambezi River system revealed *Chiloglanis* specimens that were distinguishable from the known species of the genus from southern Africa. Integration of molecular and morphological data indicated that these specimens from the Mukwadzi River represent a new species to science, herein described as *Chiloglaniscarnatus* Mutizwa, Bragança & Chakona, **sp. nov.** This species is readily distinguished from its southern African congeners by the possession of a distinctive extended dermal tissue covering the base of the dorsal fin and the possession of ten mandibular teeth (vs 8, 12, or 14 in the other taxa). Results from this study add to the growing evidence of a high level of undocumented diversity within riffle-dwelling taxa in southern Africa.

## ﻿Introduction

Rheophilic habitats are characterised by fast flowing water and rocky substratum, which provide a wide range of specialised niches for distinct aquatic taxa adapted to these environments (Thompson 2013; Hrbek et al. 2018). Delimitation of species boundaries in rheophilic taxa using only morphological traits has previously presented challenges due to their superficially similar morphology, which is shaped by exposure to similar environmental drivers (Seegers 2008). However, integrative taxonomy as well as recent collections in under-sampled areas within the African continent have changed the previous perception that rheophilic habitats were depauperate (Schmidt et al. 2015, 2016, 2017, 2023; Thomson et al. 2015; Schmidt and Barrientos 2019; Kashindye et al. 2021; Mazungula and Chakona 2021; Day et al. 2023). These studies, which implemented integrative taxonomic approaches, have allowed the discovery of hidden diversity, particularly within the catfish genera *Chiloglanis* Peters, 1868 and *Amphilius* Günther, 1864, from different regions of the continent. An emerging pattern shows that species that were previously perceived to have broad geographic ranges represent species complexes comprising distinct lineages confined to specific river basins (Chakona et al. 2018; Mutizwa et al. 2021). Recently, a careful examination of the once broadly distributed catfish species, *C.occidentalis* Pellegrin, 1933 and *C.micropogon* Poll, 1952 from West Africa and *A.natalensis* Boulenger, 1917 from southern Africa, resulted in the description of 15 new species (Schmidt et al. 2017, 2023; Mazungula and Chakona 2021). Following these findings, rheophilic habitats have been identified as a new frontier for the discovery of hidden diversity of freshwater fishes in southern Africa and other poorly explored regions on the continent (Morris et al. 2016; Schmidt et al. 2016; Chakona et al. 2018).

**Figure 1.**
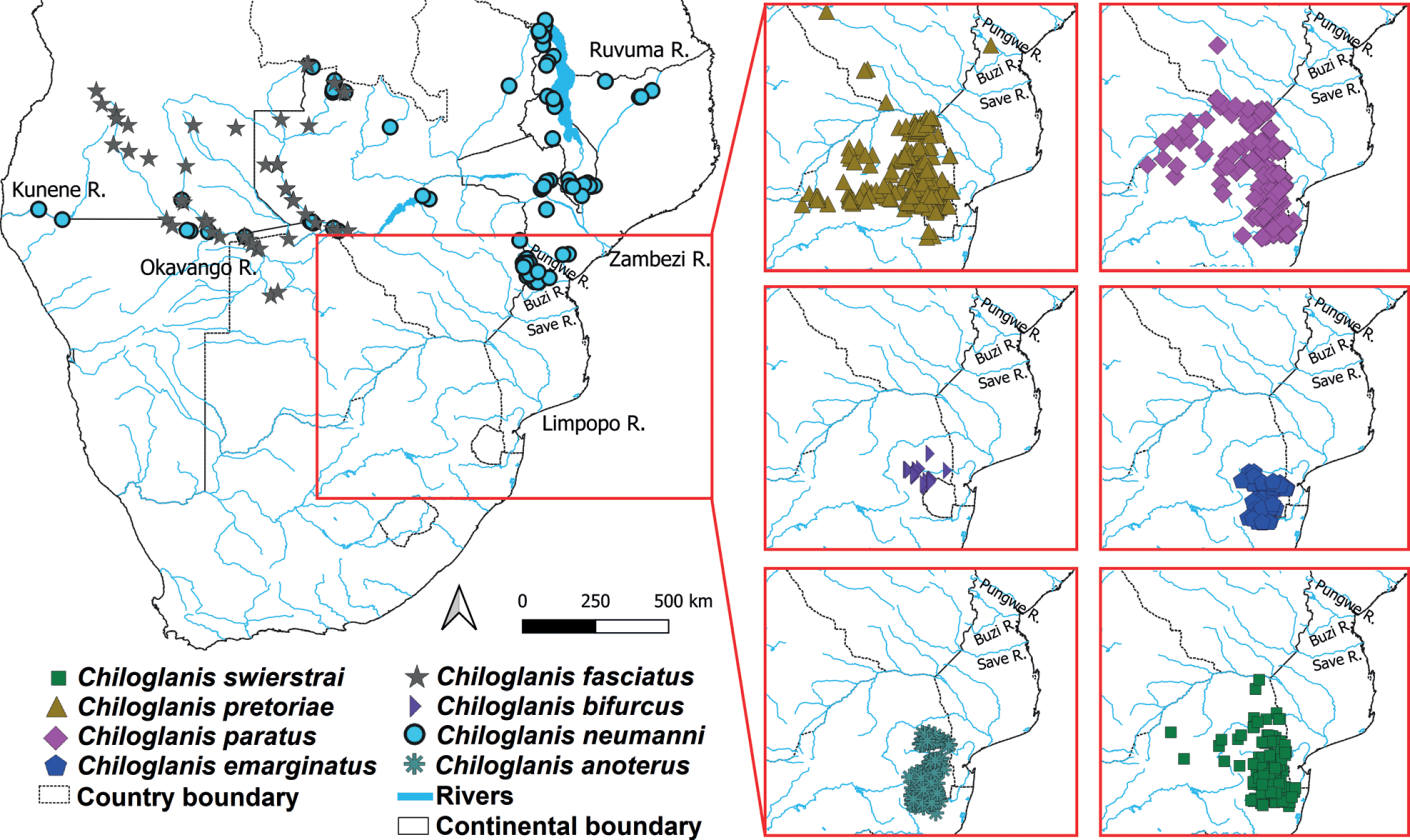
Distribution of *Chiloglanis* species in southern Africa based on data from the National Research Foundation-South African Institute for Aquatic Biodiversity extracted from the GIBF database (https://www.gbif.org).

The Mochokidae is the most species-rich freshwater catfish family that is endemic to Africa (Vigliotta 2008). Currently, this family has 228 valid species that are distributed across several river systems in sub-Saharan Africa, with the highest diversity occurring in the Congo River (Seegers 2008; Vigliotta 2008; Fricke et al. 2024). The Mochokidae is sister to a clade containing families Auchenoglanididae, Claroteidae, Malapteruridae, and Schilbeidae (Sullivan et al. 2006; Schedel et al. 2022). The genera within Mochokidae have been split into two subfamilies: the first is Chiloglanidinae, characterised by lips and barbels that are modified into an oral disc (suckermouth), a structure that is absent in the second subfamily Mochokinae. Chiloglanidinae contains the genera *Chiloglanis* Peters, 1868, *Atopodontus* Friel & Vigliotta, 2008, *Atopochilus* Sauvage, 1879, and *Euchilichthys* Boulenger, 1900, whereas Mochokinae includes the genera *Mochokus* Joannis, 1835, *Mochokiella* Howes, 1980, *Acanthocleithron* Nichols & Griscom, 1917, *Microsynodontis* Boulenger, 1903, and *Synodontis* Cuvier, 1816. Some of the intergeneric (e.g., the monophyly of Mochokinae) and the intrageneric (e.g., the monophyly of *Synodontis*) relationships within Mochokidae are not well supported and require broader species sampling to resolve (Sullivan et al. 2006; Vigliotta 2008; Day et al. 2013; Pinton et al. 2013; Schedel et al. 2022). Currently, in southern Africa *Chiloglanis* has eight recognised species: *C.bifurcus* Jubb & Le Roux, 1969, *C.emarginatus* Jubb & Le Roux, 1969, *C.anoterus* Crass, 1960, *C.paratus* Crass, 1960, *C.fasciatus* Pellegrin, 1936, *C.pretoriae* Van der Horst, 1931, *C.swierstrai* Van der Horst, 1931, and *C.neumanni* Boulenger, 1911. Except for *C.neumanni*, all these species are narrow range endemics (Fig. 1). For example, *C.bifurcus* is confined to a relatively small geographical range, occurring between 900 and 1200 metres above sea level in the Inkomati River system (Roux and Hoffman 2017a).

Uncertainties about the identity of the broadly distributed *C.neumanni* in southern Africa have persisted for decades. This species was described from the Bubu River, a tributary of the Great Ruaha River basin in Tanzania, and was considered to be distributed across several eastern, central, and southern African river systems (Daget et al. 1986; Bell-Cross and Minshull 1988). However, following extensive surveys of river systems in east Africa and comprehensive examination of specimens from this region, Seegers (1996) did not record *C.neumanni* from localities outside the Great Ruaha River system, indicating that this species was not as widely distributed as previously thought. Although the name *C.neumanni* has persisted in subsequent literature from southern Africa, ichthyologists have consistently made remarks that the suckermouth catfishes of this region required detailed taxonomic investigation to determine their identity (Marshall 2011). In recent years, there has been general consensus among southern African ichthyologists that the species currently referred to as *C.neumanni* in this region actually represents an undescribed species or even a species complex, including several undescribed species. This assertion is based on the extensive geographic distance between southern Africa and the Bubu River, as well as the emerging patterns of undescribed diversity within other species with similar distribution ranges as *C.neumanni*. For example, studies of *A.uranoscopus* (Pfeffer, 1889) and *Zaireichthysrotundiceps* (Hilgendorf, 1905) led to the resurrection of two synonyms and the description of nine new species (Thomson and Page 2010; Eccles et al. 2011). Indeed, a genetic study by Chakona et al. (2018) identified six unique lineages within the genus *Chiloglanis* from the Eastern Zimbabwe Highlands ecoregion, a result that is consistent with Marshall’s (2011) postulation that the continued use of the name *C.neumanni* in southern Africa potentially obscures the actual diversity of this group of fishes in this region. A total of four new species to science are currently being described from the Eastern Zimbabwe Highlands ecoregion, with two of them being endemic to this region (Chakona et al., pers. obs.).

During surveys of the southern tributaries of the middle Zambezi River system in 2016 and 2019, morphologically distinct suckermouth catfishes were collected from the Mukwadzi River that drains the western margin of the Great Dyke in Zimbabwe. These specimens could not be attributed to any of the currently described species or recently identified lineages of *Chiloglanis* from this region. The present work represents the first in a series of studies that aim to resolve the taxonomy of suckermouth catfishes of southern Africa. This study applied integrative taxonomic approaches combining genetic and morphological data to determine the taxonomic distinctiveness of the recently collected specimens from the middle Zambezi River. The significance and implications of incomplete documentation of the diversity of rheophilic species in a region where their unique habitats are under threat from multiple environmental impacts are discussed.

## ﻿Materials and methods

### ﻿Collections

Specimens were collected from two sites in the Mukwadzi River, a tributary of the Manyame River, a south bank affluent of the middle Zambezi River, during surveys in 2016 and 2019. Samples were collected using a battery-powered Samus 725GN backpack electric fisher with a block net placed downstream to capture dislodged animals in the fast-flowing current. The specimens were photographed to document the live colour pattern then euthanized with clove oil. Muscle tissue from the right side of the specimens was cut out and preserved in 99% ethanol for molecular analysis. Voucher specimens for morphological studies were fixed in 10% formalin in the field and subsequently transferred to 70% ethanol for long term preservation. Additional tissue samples and voucher specimens used in the present study were obtained from the National Fish Collection at the National Research Foundation-South African Institute for Aquatic Biodiversity (NRF-SAIAB) in Makhanda (Tables 1, 2).

**Table 1.** List of 80 COI sequences used in the present study including six new sequences of the specimens from the Mukwadzi River. The new sequences (in bold) include the hologenetype identified by an asterisk (*) and paragenetypes identified by a plus (+).

Species name	River system	GPS coordinates (Latitude, Longitude)	COI sequence ID
*Atopochilussavorgnani*	Congo	–	MK073983
	Congo	–	MK073984
*Chiloglanisanoterus*	Mlumati	-25.7567, 31.4386	LN610269
	Mlumati	-25.7692, 31.3367	LN610270
	Mlumati	-25.7692, 31.3367	LN610271
	Mlumati	-25.8672, 31.3347	LN610272
*Chiloglanisbifurcus*	Mlumati	–	MH432062
	Mlumati	–	SB8458
	Mlumati	–	SB8462
*Chiloglanisfasciatus*	Okavango	-13.5943, 16.8805	ANGFW077-12
	Okavango	-12.6713, 16.1114	ANGFW131-12
	Okavango	-12.6713, 16.1114	ANGFW132-12
	Okavango	-12.6713, 16.1114	ANGFW133-12
	Okavango	-12.6713, 16.1114	ANGFW134-12
*Chiloglanisparatus*	Phongolo	–	MPUMA025
	Phongolo	–	SB8459
*Chiloglanispretoriae*	Limpopo	-23.9904, 31.8258	LN610341
*Chiloglanis* sp. ‘dwarf’	Honde	-18.4337, 32.8969	MH432047
	Honde	-18.5992, 32.729	MH432054
	Makanga	-18.5438, 32.8013	MH432044
	Mupenga	-18.5725, 32.8038	MH432042
	Mupenga	-18.5725, 32.8038	MH432048
	Mutarazi	-18.5324, 32.8075	MH432018
*Chiloglanis* sp. ‘dwarf’	Mutarazi	-18.5324, 32.8075	MH432019
	Mutarazi	-18.5324, 32.8075	MH432032
	Nyamhingura	-18.3696, 32.9354	MH432025
	Nyamhingura	-18.3696, 32.9354	MH432026
	Nyamhingura	-18.3696, 32.9354	MH432027
	Phalombe	-15.81, 35.646	MAFW097
	Pungwe	-18.3955, 32.9707	MH432030
	Pungwe	-18.3955, 32.9707	MH432031
	Pungwe	-18.45, 32.8968	MH432046
	Pungwe	-18.45, 32.8968	MH432057
	Pungwe	-18.3955, 32.9707	MH432061
	Ruo	-16.0403, 35.6633	MAFW029
*Chiloglanis* sp. ‘Shire’	Shire	-15.061, 35.219	MAFW119
***Chiloglaniscarnatus* sp. nov.**	**Manyame**	** -17.4249, 30.5854 **	PP156890*
	**Manyame**	** -17.4249, 30.5854 **	**PP156891 ^+^**
	**Manyame**	** -17.4249, 30.5854 **	**PP156892 ^+^**
	**Manyame**	** -17.4249, 30.5854 **	**PP156893 ^+^**
	**Manyame**	** -17.4249, 30.5854 **	**PP156894 ^+^**
	**Manyame**	** -17.4249, 30.5854 **	**PP156895 ^+^**
*Chiloglanis* sp. ‘Nyangombe’	Chidya	-18.2653, 32.5903	MH432020
	Chidya	-18.2653, 32.5903	MH432021
	Chidya	-18.2653, 32.5903	MH432022
	Chidya	-18.2653, 32.5903	MH432033
*Chiloglanis* sp. ‘Pungwe’	Chiyengwa	-18.6878, 32.922	MH432040
	Honde	-18.5992, 32.729	MH432049
	Pungwe	-18.3955, 32.9707	MH432028
	Pungwe	-18.3955, 32.9707	MH432029
*Chiloglanis* sp. ‘roughskin’	Buzi	-19.932, 33.826	SAFW910
	Chiyengwa	-18.6878, 32.922	MH432045
	Chiyengwa	-18.6878, 32.922	MH432051
	Honde	-18.5438, 32.8044	MH432036
	Makanga	-18.5438, 32.8013	MH432043
	Mupenga	-18.5725, 32.8038	MH432038
	Mupenga	-18.5725, 32.8038	MH432039
	Mupenga	-18.5725, 32.8038	MH432041
	Mupenga	-18.5725, 32.8038	MH432060
	Ngarura	-18.5474, 32.8718	MH432052
	Ngarura	-18.5474, 32.8718	MH432053
	Ngarura	-18.5474, 32.8718	MH432059
	Nyamukombe	-18.3821, 33.0327	MH432034
	Nyamukombe	-18.3821, 33.0327	MH432035
	Nyamukombe	-18.3821, 33.0327	MH432058
	Nyamukwara	-18.6918, 32.9236	MH432055
	Nyamukwara	-18.6918, 32.9236	MH432056
	Pungwe	-18.4414, 32.8875	MH432050
	Rwera	-18.5434, 32.8044	MH432037
*Chiloglanis* sp. ‘Zambezi’	Zambezi	-15.656, 30.953	SAFW893
	Nyangombe	-18.0829, 32.5819	MH432023
	Nyangombe	-18.0829, 32.5819	MH432024
	Okavango	-14.9397, 17.7188	ANGFW015-12
	Okavango	-13.5943, 16.8805	ANGFW078-12
	Okavango	-14.6497, 16.9066	ANGFW211-12
*Chiloglanisswierstrai*	Phongolo	–	SB8457
	Phongolo	–	SB8460
	Phongolo	–	SB8461
*Euchilichthysboulengeri*	Dipumu	-6.0045, 22.3905	HM418085
*Euchilichthysroyauxi*	Epulu	–	KT192823

**Table 2.** List of 184 specimens examined in this study including 19 specimens collected from the Mukwadzi River.

Species	Type status	Catalogue No.	No. specimens	River system	Latitude, Longitude
*Chiloglanisanoterus*	Holotype	SAIAB 186246	1	Phongola	-27.5, 30.4667
*Chiloglanisbifurcus*	Holotype	SAIAB 120160	1	Incomati	-25.4333, 30.7167
	Paratype	SAIAB 120161	6	Incomati	-25.4333, 30.7167
	Paratype	SAIAB 120529	3	Incomati	-25.3833, 30.35
*Chiloglanisemarginatus*	Holotype	SAIAB 120117	1	Incomati	-25.9833, 30.6833
	Paratype	SAIAB 120118	9	Incomati	-25.85, 30.2
*Chiloglanisfasciatus*	_	SAIAB 204928	6	Okavango	-14.3872, 16.2876
	_	SAIAB 204916	4	Okavango	-14.387, 16.2873
***Chiloglaniscarnatus* sp. nov.**	**Holotype**	**SAIAB 236631**	**1**	**Manyame**	** -17.4249, 30.5854 **
	**Paratype**	**SAIAB 211349**	**13**	**Manyame**	** -17.4244, 30.5845 **
	**Paratype**	**SAIAB 211346**	**5**	**Manyame**	** -17.4249, 30.5854 **
*Chiloglanisparatus*	Holotype	SAIAB 186248	1	Phongola	-27.3833, 31.5
	Paratype	SAIAB 120050	1	Incomati	_
*Chiloglanisswierstrai*	Paratype	SAIAB 30013	1	Phongola	-25.6667, 27.8333
	Paratype	SAIAB 21805	5	Phongola	-27.4333, 31.5167
	Holotype	SAIAB 186247	1	Phongola	-27.4167, 31.1833
*Chiloglanispretoriae*	_	SAIAB 82972	10	Limpopo	-23.0105, 30.4785
	_	SAIAB 70603	3	Incomati	-25.8478, 27.7836
	_	SAIAB 70822	3	Limpopo	-25.3883, 28.3117
*Chiloglanisneumanni*	Lectotype	BMNH190575249	1	Bubu	_
	Paralectotype	BMNH190575250	1	Bubu	_
	Paralectotype	BMNH190575250	1	Bubu	_
*Chiloglanis* sp. ‘rough skin’	_	SAIAB 201075	4	Pungwe	-18.4414, 32.8875
	_	SAIAB 201095	2	Chiyengwa	-18.6878, 32.922
	_	AC14CL10	11	Mupenga	-18.5725, 32.8038
	_	SAIAB 200955	5	Ngarura	-18.5474, 32.8718
	_	SAIAB 200933	9	Nyamukombe	-18.3821, 33.0327
	_	SAIAB 201035	15	Rwera	-18.5434, 32.8044
	_	SAIAB 201047	3	Nyamukombe	-18.3821, 33.0327
	_	SAIAB 201088	8	Nyamukwara	-18.6918, 32.9236
	_	SAIAB 201026	8	Honde	-18.5438, 32.8044
*Chiloglanis* sp. ‘dwarf’	_	AC14CL10	10	Mupenga	-18.5725, 32.8038
	_	SAIAB 200940	3	Pungwe	-18.45, 32.8968
	_	SAIAB 200923	1	Pungwe	-18.3955, 32.9707
	_	SAIAB 205087	5	Mutarazi	-18.5324, 32.8075
	_	SAIAB 205074	3	Nyamhingura	-18.3696, 32.9354
	_	AC13BL04	3	Pungwe	-18.3955, 32.9707
*Chiloglanis* sp. ‘Pungwe’	_	AC13BL04	2	Pungwe	-18.3955, 32.9707
	_	SAIAB 201095	1	Chiyengwa	-18.6878, 32.922
	_	SAIAB 201067	1	Honde	-18.5992, 32.729
*Chiloglanis* sp. ‘Nyangombe’	_	SAIAB 210408	6	Chidya	-18.2653, 32.5903
*Chiloglanis* sp. ‘Zambezi’	_	SAIAB 200517	2	Nyangombe	-18.0829, 32.5819
	_	SAIAB 81243	2	Lower Zambezi	-15.656, 30.953
	_	SAIAB 186643	1	Okavango	-14.9397, 17.7188
	_	SAIAB 186709	1	Okavango	-13.5943, 16.8805

### ﻿DNA extraction, amplification, and sequencing

A total of six new COI sequences of *Chiloglaniscarnatus* sp. nov. were generated for this study. Preparation and sequencing of genetic material was done in the Aquatic Genomics Research Platform at the NRF-SAIAB. Genomic DNA was extracted from preserved tissues using the salting-out method (Sunnucks and Hales 1996). The mitochondrial DNA cytochrome c oxidase subunit I (COI) gene was amplified by polymerase chain reaction (PCR) using the universal fish DNA barcoding primer set FishF1 and FishR1 (Ward et al. 2005). PCRs were performed with a Veriti 96 well thermal cycler (Applied Biosystems, USA) and each reaction mixture (25 µL) contained 50–100 ng) of template DNA, 6.5 µL of water, 0.5 µL of each primer (10 µM), and 12.5 µL Taq DNA polymerase 2× master mix red (Amplicon PCR enzymes and reagents, Denmark). The PCR amplification profile had an initial denaturation step of 3 min at 94 °C followed by 38 cycles of 30 sec at 94 °C, annealing at 55 °C for 30 sec, and extension at 72 °C for 50 sec, and final extension at 72 °C for 7 min. The amplicons were purified using an Exonuclease I-Shrimp Alkaline Phosphate (Exo/SAP, Thermo Fisher Scientific, USA) protocol (Werle et al. 1994), sequenced using standard fluorescent BigDye v. 3.1 (Applied Biosystems, USA) terminator chemistry in the forward direction, and analysed on a 3500 Genetic Analyser (Applied Biosystems, USA) at the NRF-SAIAB. Additional sequences were obtained from the public databases GenBank (https://www.ncbi.nlm.nih.gov/genbank/) and Barcode of Life Data Systems (BOLD) (http://www.boldsystems.org/) (Table 1).

### ﻿Phylogenetic analyses

Phylogenetic analyses included genetic sequences generated from *Chiloglaniscarnatus* sp. nov., six of the seven nominal species from southern Africa, six candidate species of *Chiloglanis* identified by Chakona et al. (2018) (*Chiloglanis* sp. ‘roughskin’, *Chiloglanis* sp. ‘Zambezi’, *Chiloglanis* sp. ‘Nyangombe’, *Chiloglanis* sp. ‘Pungwe’, *Chiloglanis* sp. ‘Shire’, *Chiloglanis* sp. ‘dwarf’), and three outgroup species (*Euchilichthysboulengeri* Nichols & LaMonte, 1934; *Euchilichthysroyauxi* Boulenger, 1902; *Atopochilussavorgnani* Sauvage, 1879) (Table 1). Genetic material for *C.neumanni* from its type locality and *C.emarginatus* could not be accessed before finalising this study. Mitochondrial DNA sequences were edited, aligned, and trimmed in MEGA-X (Kumar et al. 2016). The sequences were translated into amino acid sequences in MEGA-X to check for stop codons and gaps to ensure that they were copies of functional mitochondrial protein coding sequences. Haplotype groups were identified using DNASP 6 (Rozas et al. 2017). The most suitable model for nucleotide substitution was selected using the Akaike Information Criterion (AIC) (Akaike 1974) as implemented in the program jModelTest 0.1.1 (Darriba et al. 2012). Bayesian phylogenetic inference was performed in MrBayes 3.2.6 (Ronquist et al. 2012) using the TIM3+I+G evolutionary model identified using jModeltest. The phylogenetic tree and posterior probabilities were inferred using four Markov chain Monte Carlo (MCMC) chains which were run for 2 × 10^6^ generations with tree sampling every 1000 generations. The program Tracer 1.7 (Rambaut et al. 2018) was used to analyse the quality of the trace files generated by MrBayes and to determine the number of trees to be discarded as burn-in. The first 25% of the sampled trees for each analysis was discarded as burn-in, and the remaining trees were used to calculate a majority rule consensus tree. Maximum likelihood (ML) analysis of the same dataset was performed in RAxML v. 8 (Stamatakis 2014) through the graphical user interface raxmlGUI v. 2 (Silvestro and Michalak 2012). A total of 10 ML searches were performed in raxmlGUI and support values for the ML tree nodes were estimated by 1000 non-parametric bootstrap inferences (Felsenstein 1985). Bootstrap values equal to or higher that 70% (Hillis and Bull 1993), and posterior probability values at 0.95 or higher (Alfaro and Holder 2006), were considered strong support.

### ﻿Molecular species delimitation

Four molecular species delimitation methods were used to delineate candidate species within the suckermouth catfishes of southern Africa using the same dataset used for the phylogenetic analysis. The first two methods, Automatic Barcode Gap Discovery (ABGD; Puillandre et al. 2012) and Assemble Species by Automatic Partitioning (ASAP; Puillandre et al. 2021) infer the barcode gap from the data to partition sequences into proposed candidate species. These methods were performed on their respective webservers (https://bioinfo.mnhn.fr/abi/public/abgd/ and https://bioinfo.mnhn.fr/abi/public/asap/asapweb.html). The intraspecific diversity priors were set at P_min_ = 0.001 and P_max_ = 0.1) for both methods. The Kimura (K80) TS/TV distance model was used and the remaining settings were left at their default parameters. The second pair of species delimitation methods included the Bayesian implementation of the Poisson Tree Processes (bPTP) (Zhang et al. 2013) and the General Mixed Yule Coalescent (GMYC) (Pons et al. 2006; Fujisawa and Barraclough 2013). Both GMYC and bPTP require a phylogenetic tree as input and from this tree they estimate rates of branching events to infer which parts of the tree are likely to follow a speciation model (interspecific variation) and which parts follow a coalescent model (intraspecific variation). The bPTP was performed on the web server (http://species.h-its.org/ptp/) using the same tree generated for phylogenetic reconstruction and a MCMC run for 1 × 10^6^ generations with 10% burn-in. For the GMYC analysis a fully resolved ultrametric tree was inferred in Bayesian evolutionary analysis by sampling trees (BEAST) 2.4.6 (Bouckaert et al. 2014) using a strict clock and Yule model and the MCMC was ran for 1 × 10^7^ generations with tree sampling every 1000 generations. The program Tracer 1.7.2 was used to analyse the quality of the trace files generated by BEAST. TreeAnnotator (Helfrich et al. 2018) was used to summarise the trees sampled by BEAST into a single maximum credibility tree with a burn-in of 25%. The species’ limits by threshold Statistics (splits) package (http://r-forge.r-project.org/projects/splits) in R 3.5.0 (R Core Team 2018) was used to identify the candidate species from the maximum credibility tree produced by TreeAnnotator. Model corrected intraspecific and interspecific genetic distances of the molecular taxonomic units identified by the species delimitation methods were calculated in PAUP* 4.0a163 (Swofford 2003).

### ﻿Morphological analyses

**Figure 2.**
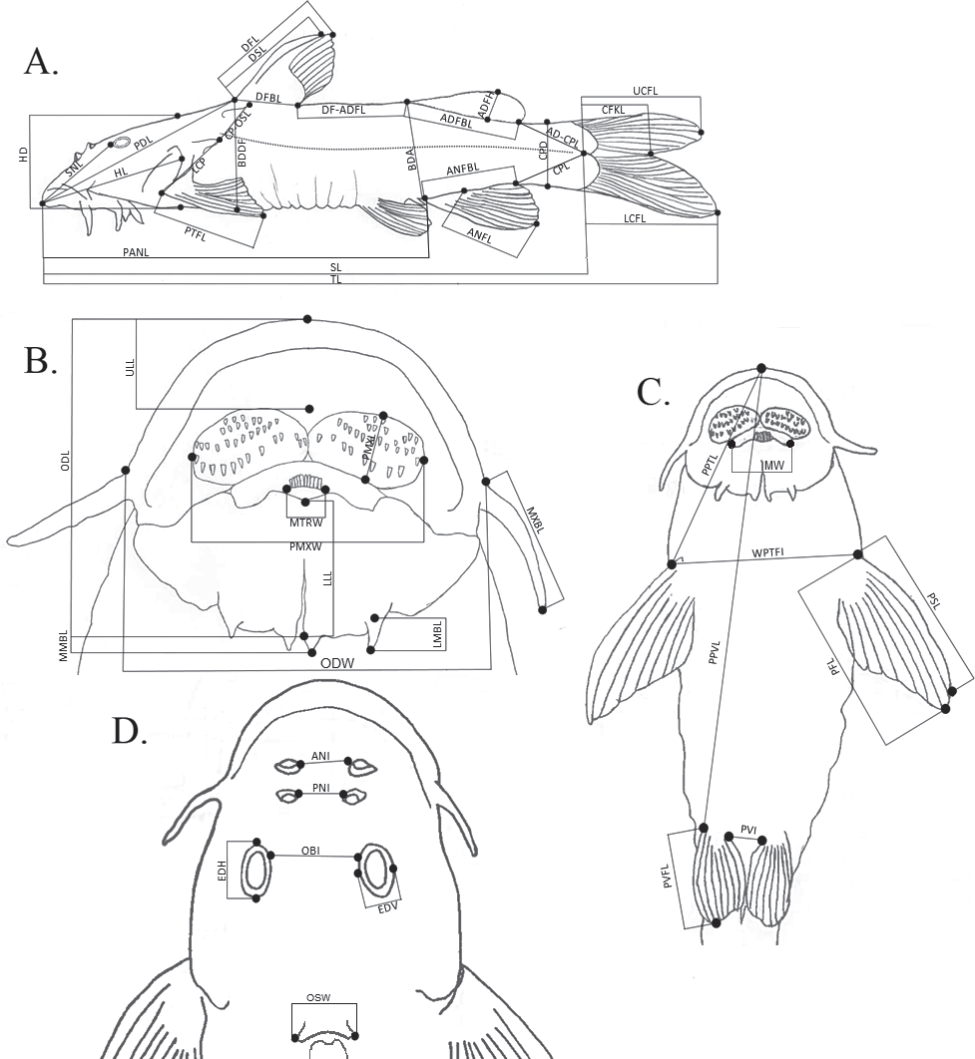
Illustrations depicting linear measurements recorded from *Chiloglanis* specimens **A** lateral view **B** ventral view of the Oral disc **C** ventral view **D** dorsal view of the head. Abbreviations: AD-CPL-adipose fin to caudal peduncle length, ADFBL-adipose-fin base length, ADFH-adipose-fin height, ANFBL-anal-fin base length, ANFL-anal-fin length along longest ray, ANI-anterior nares interspace, BDA-body depth at anus, BDDF-body depth at dorsal-fin insertion, CFKL-caudal fork length, CPD-caudal peduncle depth, CPL-caudal peduncle length, CP-OSL- post-cleithral process to occipital shield length, DF-ADFL-dorsal fin to adipose fin length, DFBL-dorsal-fin base length, DFL-dorsal-fin length along longest ray, DSL-dorsal-spine length, EDH-eye diameter (horizontal axis), EDV-eye diameter (vertical axis), HD-head depth, HL-head length to opercular membrane margin, LCFL-Lower caudal-fin lobe length, LCP-length of post-cleithral process, LLL-lower lip length, LMBL-Lateral mandibular barbel length, MMBL-Medial mandibular barbel length, MTRW-mandibular tooth row width, MXBL-maxillary barbel length, MW-mouth width, OBI-orbital interspace, ODL-oral disc length, ODW-oral disc width, OSW-occipital shield width, PANL-pre-anal length, PDL-pre-dorsal length, PMXL-pre-maxillary tooth-patch length, PMXW- pre-maxillary tooth patch width, PNI-posterior nares interspace, PPTL-pre-pectoral length, PPVL-pre-pelvic length, PSL-pectoral-spine length, PFL-pectoral-fin length, PVFL-pelvic-fin length, PVI-pelvic-fin interspace, SL-standard length, SNL-snout length, TL-total length, UCFL–Upper caudal-fin lobe length, ULL-upper lip length, WPTFI-width at pectoral-fin insertion.

**Figure 3.**
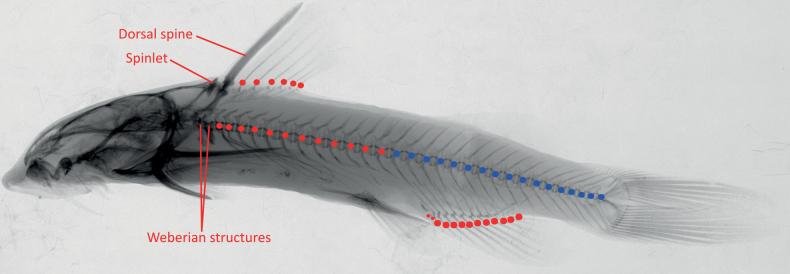
Illustration showing how fin rays and vertebrae were counted using x-ray radiographs. The red dots along the vertebra represent the abdominal vertebrae and the blue dots represent the caudal vertebrae.

A total of 19 specimens of *Chiloglaniscarnatus* sp. nov. collected from the Mukwadzi River were examined in the present study. Comparative material included the lectotype of *C.neumanni*, holotypes of six valid species from southern Africa and five candidate species identified by Chakona et al. (2018) (Table 2). Because type material for *C.fasciatus* could not be accessed before finalising this study, 10 conspecific specimens collected from close to the type locality of this species and identified using the species description by Pellegrin (1936) and the key in Skelton (2001) were used as topotypes. The syntypes of *C.pretoriae* were severely deformed, thus only their meristic counts were included for comparison in this study, but 16 specimens collected from near the type locality of *C.pretoriae* and identified using the key in Skelton (2001) were used as topotypes for this species. Formulae and terminology of morphometric and meristic characters followed Schmidt et al. (2015), Friel and Vigliotta (2008), and Skelton and White (1990). A total of 49 morphometric characters were measured to the nearest 0.1 mm using digital Vernier callipers following Friel and Vigliotta (2008) (Table 3, Fig. 2A–D). External meristic counts were performed under a stereo microscope. Vertebrae counts were made from radiographs taken at the NRF-SAIAB using an Inspex 20i Digital X-ray Imaging System (Kodex Inc., New Jersey, USA). Radiographs for the lectotype and paralectotypes of *C.neumanni* were taken at the Royal Museum for Central Africa, Belgium (MRAC) using a VisiX-MedexLoncin (www.medex.be). A total of nine meristic characters were examined: number of mandibular teeth, pre-maxillary teeth, pectoral-fin rays, pelvic-fin rays, dorsal-fin rays, anal-fin rays, abdominal vertebrae, caudal vertebrae, and total vertebrae (Fig. 3). Following Roberts (1989), vertebrae counts excluded the Weberian structures and began from the fifth vertebra which was identified by a pair of large but slender ribs, and included the hypural complex which was counted as one vertebra. The abdominal vertebrae were defined as the vertebrae that occurred anterior to the first anal fin ray pterygiophore. Caudal vertebrae were defined as those that occurred posterior to the first anal fin ray pterygiophore and included the hypural complex which was counted as one vertebra (Roberts 1989) (Fig. 3). The genital papillae were examined to determine the sex of the specimens following Friel and Vigliotta (2008). Morphological measurements were standardised by transforming body measurements into percentages of the standard length (**SL**) and head measurements into percentages of the head length (**HL**). Principal component analyses (PCA) were performed in PAST v. 3.12 (Hammer et al. 2001) using the covariance matrix for the morphometric data in order to identify morphological characters that contributed the most to distinguishing *Chiloglaniscarnatus* sp. nov. from the other *Chiloglanis* species from southern Africa.

**Table 3.** Morphological characters examined in the present study.

Morphological characters	Abbreviation
Adipose fin to caudal peduncle length	AD-CPL
Adipose-fin base length	ADFBL
Adipose-fin height	ADFH
Anal-fin base length	ANFBL
Anal-fin length along longest ray	ANFL
Anterior nare interspace	ANI
Body depth at anus	BDA
Body depth at dorsal-fin insertion	BDDF
Caudal fork length	CFKL
Caudal peduncle depth	CPD
Caudal peduncle length	CPL
Dorsal fin to adipose fin length	DF-ADFL
Dorsal-fin base length	DFBL
Dorsal-fin length along longest ray	DFL
Dorsal-spine length	DSL
Eye diameter (horizontal axis)	EDH
Eye diameter (vertical axis)	EDV
Head depth	HD
Head length to opercular membrane margin	HL
Lateral mandibular barbel length	LMBL
Length of post-cleithral process	LCP
Lower caudal-fin lobe length	LCFL
Lower lip length	LLL
Mandibular tooth row width	MTRW
Maxillary barbel length	MXBL
Medial mandibular barbel length	MMBL
Mouth width	MW
Occipital shield width	OSW
Oral disc length	ODL
Oral disc width	ODW
Orbital interspace	OBI
Pectoral-fin length	PFL
Pectoral-spine length	PSL
Pelvic-fin interspace	PVI
Pelvic-fin length	PVFL
Post-cleithral process to occipital shield length	CP-OSL
Posterior nares interspace	PNI
Pre-anal length	PANL
Pre-dorsal length	PDL
Pre-maxillary tooth-patch length	PMXL
Pre-maxillary tooth-patch width	PMXW
Pre-pectoral length	PPTL
Pre-pelvic length	PPVL
Snout length	SNL
Standard length	SL
Total length	TL
Upper caudal-fin lobe length	UCFL
Upper lip length	ULL
Width at pectoral-fin insertion	WPTFI
Abdominal vertebrae	–
Anal fin-ray count	–
Caudal vertebrae	–
Dorsal fin-ray count	–
Mandibular tooth count	–
Pectoral fin-ray count	–
Pelvic fin-ray count	–
Pre-maxillary tooth count	–
Total vertebrae	–

## ﻿Results

### ﻿Phylogenetic analyses

**Figure 4.**
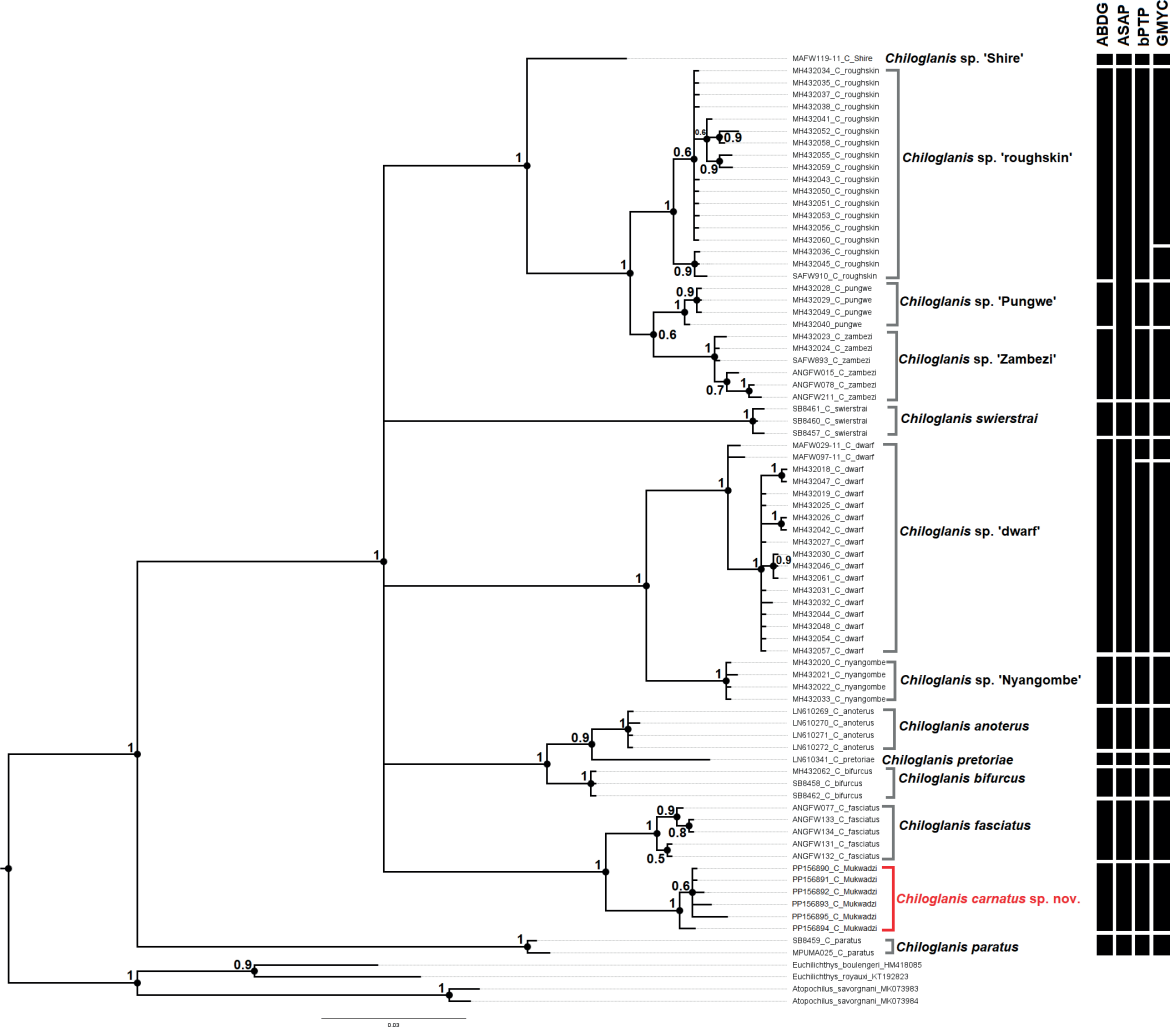
Bayesian inference tree of the species and lineages of the genus *Chiloglanis* found in southern African. The numbers at the nodes represent the Bayesian posterior probabilities. The black bars represent candidate species proposed by four molecular species delimitation methods: Automatic Barcode Gap Discovery (ABGD), Automatic Partitioning (ASAP), Bayesian implementation of the Poisson Tree Processes (bPTP), and General Mixed Yule Coalescent (GMYC).

**Figure 5.**
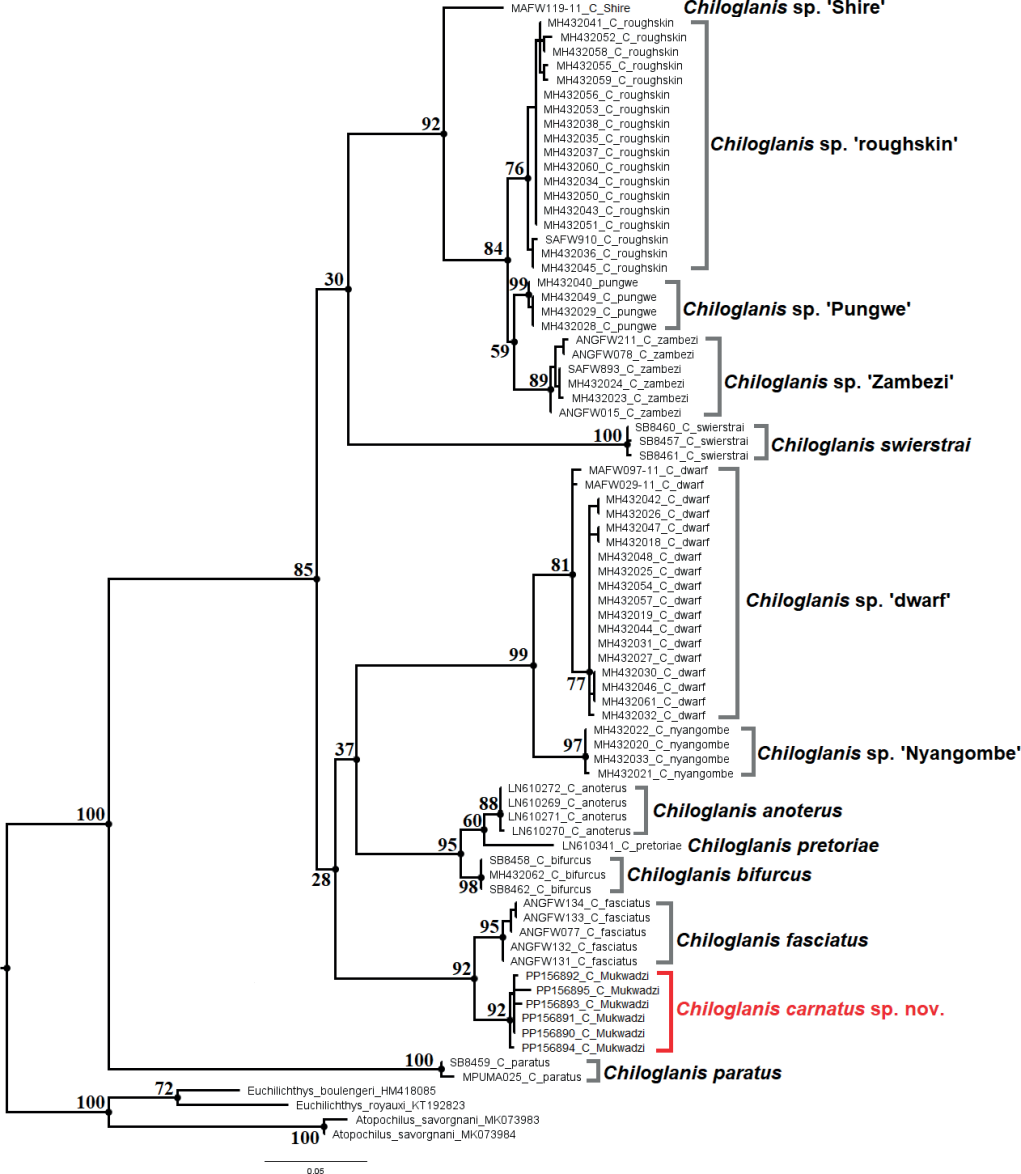
Maximum likelihood tree of the species and lineages of the genus *Chiloglanis* found in southern African. The numbers at the nodes represent the bootstrap values.

The COI alignment of 80 sequences had 534 base pairs and 176 variable sites. A total of 47 unique haplotypes were identified. Although the Bayesian phylogenetic tree was not fully resolved, it showed genetic structuring that supported the monophyly of suckermouth catfishes from southern Africa (Fig. 4). *Chiloglaniscarnatus* sp. nov. was recovered as an exclusive group that is genetically divergent (2.8–15.0% genetic distances) from other *Chiloglanis* species and lineages from southern Africa (Figs 4, 5; Table 4). With the exception of *C.pretoriae* and *Chiloglanis* sp. ‘Shire’, all recovered clades were well-supported (posterior probability > 0.95). Genetic divergences within valid and candidate species ranged from 0–1.5% and interspecific divergences ranged from 1.3–15.7% (Table 4). *Chiloglanisparatus* from the Phongolo River was recovered as the most basal clade that is sister species to all the southern African suckermouth catfishes included in the present study. The relationships among the remaining taxa were not well resolved as they were recovered within five polytomous clades with weak support between them (Fig. 4). The first clade contained *Chiloglaniscarnatus* sp. nov. from the Manyame River and *C.fasciatus* from the Okavango River. The second clade contained *C.anoterus* and *C.bifurcus* from the Incomati River system as well as *C.pretoriae* from the Limpopo River system. The third clade contained *Chiloglanis* sp. ‘Nyangombe’ from the Nyangombe River and *Chiloglanis* sp. ‘dwarf’ from the Pungwe and Ruo rivers. The fourth clade contained *Chiloglanisswierstrai* from the Limpopo River system. The fifth clade contained the *Chiloglanis* sp. ‘Zambezi’, *Chiloglanis* sp. ‘Pungwe’, *Chiloglanis* sp. ‘roughskin’, and *Chiloglanis* sp. ‘Shire’ lineages. The *Chiloglanis* sp. ‘roughskin’ lineage occurs in the Buzi and Pungwe rivers, whereas *Chiloglanis* sp. ‘Pungwe’ is endemic to the Pungwe River. *Chiloglanis* sp. ‘Shire’ and *Chiloglanis* sp. ‘Zambezi’ lineages were found in the lower Zambezi River system with the latter lineage also occurring in the Okavango River. The phylogenetic tree inferred using the ML approach had similar topology to the Bayesian inference tree (Fig. 5).

**Table 4.** Ranges of cytochrome oxidase I (COI) genetic distances (%) between the *Chiloglanis* species included in the present study.

		1	2	3	4	5	6	7	8	9	10	11	12	13	14	15	16
1	*Chiloglanis* sp. ‘dwarf’	0–1.5															
2	*Chiloglanis* sp. ‘Nyangombe’	3.6–4.5	0–0.2														
3	*Chiloglanis* sp. ‘Zambezi’	10.7–11.4	9.0–9.7	0–0.9													
4	*Chiloglanis* sp. ‘Pungwe’	11.0–11.8	9.9–10.3	2.1–3.0	0–0.2												
5	*Chiloglanis* sp. ‘roughskin’	10.5–11.6	9.4–10.3	2.2–3.9	1.3–2.6	0–1.3											
6	*Chiloglanis* sp. ‘Shire’	11.4–11.9	10.1–10.3	5.1–5.6	5.0–5.2	4.1–5.2	_										
7	*Chiloglaniscarnatus* sp. nov.	12.0–13.7	12.9–13.9	10.7–12.0	11.0–12.0	10.1–11.2	10.3–11.0	0–1.1									
8	*Chiloglanisanoterus*	11.0–11.4	11.0–11.2	9.5–10.1	9.7–10.7	9.6–9.9	9.7	9.7–11.4	0–0.2								
9	*Chiloglanispretoriae*	9.9–10.3	10.1–10.3	10.7–11.0	10.7–10.8	11.0–11.4	10.1	10.7–11.4	3.4–3.6	_							
10	*Chiloglanisfasciatus*	10.9–12.0	12.0–12.4	10.9–11.4	11.2–11.6	10.3–10.9	10.3	2.8–3.9	9.0–9.2	9.6–9.7	0–0.6						
11	*Chiloglanisswierstrai*	12.4–13.9	13.7–14.2	11.4–11.8	11.4–11.8	11.2–11.8	10.1–11.0	12.4–13.3	11.4–11.8	12.4–12.7	9.0–12.5	0.2–0.4					
12	*Chiloglanisbifurcus*	9.9–10.3	10.5–10.7	9.9–10.3	10.5–10.7	10.3–10.7	9.4	9.8–10.5	2.4–2.6	4.1	9.0–9.2	11.8–12.2	0				
13	*Chiloglanisparatus*	15.0–15.5	14.4–14.8	14.2–14.8	14.8–15.2	15.0–15.7	13.9–14.0	14.0–15.0	13.3–13.7	13.7–13.9	13.7–14.4	15.4–15.7	13.1–13.5	0.6			
14	*Atopochilussavorgnani*	15.7–16.1	15.5–16.3	15.0–15.9	15.5–16.1	15.0–15.5	14.2–14.4	15.5–16.9	15.0–15.7	14.8–15.0	15.0–15.5	16.5–16.9	15.2–15.7	15.0–15.5	1.1		
15	*Euchilichthysboulengeri*	15.2–15.4	15.0–15.2	15.2–15.4	14.4	14.0–14.2	14.2	15.4–16.1	14.6–15.4	14.8	15.0–15.4	13.7–13.9	14.6–15.4	13.3–13.5	11.2–11.4	_	
16	*Euchilichthysroyauxi*	16.5–17.0	16.3–16.5	16.3–16.9	15.7	15.4–16.1	14.8	15.7–16.5	16.3–16.5	16.5	15.2–15.5	15.4–15.5	16.3	13.5–3.9	10.7	6.6	_

### ﻿Molecular species delimitation

All four molecular species delimitation methods identified *Chiloglaniscarnatus* sp. nov., *Chiloglanis* sp. ‘Shire’, *Chiloglanis* sp. ‘Nyangombe’, *C.swierstrai*, *C.anoterus*, *C.pretoriae*, *C.bifurcus*, *C.fasciatus*, and *C.paratus* as unique molecular taxonomic units (Fig. 4). The Assemble Species by Automatic Partitioning method recovered the least number of candidate species, this method grouped *Chiloglanis* sp. ‘roughskin’, *Chiloglanis* sp. ‘Pungwe’, and *Chiloglanis* sp. ‘Zambezi’ into a single molecular taxonomic unit. The General Mixed Yule Coalescent method recovered the highest number of molecular taxonomic units. This method identified additional molecular taxonomic units within *Chiloglanis* sp. ‘roughskin’ and *Chiloglanis* sp. ‘dwarf’. The Automatic Barcode Gap Discovery and bPTP inferred similar molecular taxonomic units with the exception of *Chiloglanis* sp. ‘dwarf’ which was split into two molecular taxonomic units by the latter method.

### ﻿Morphological analyses

**Figure 6.**
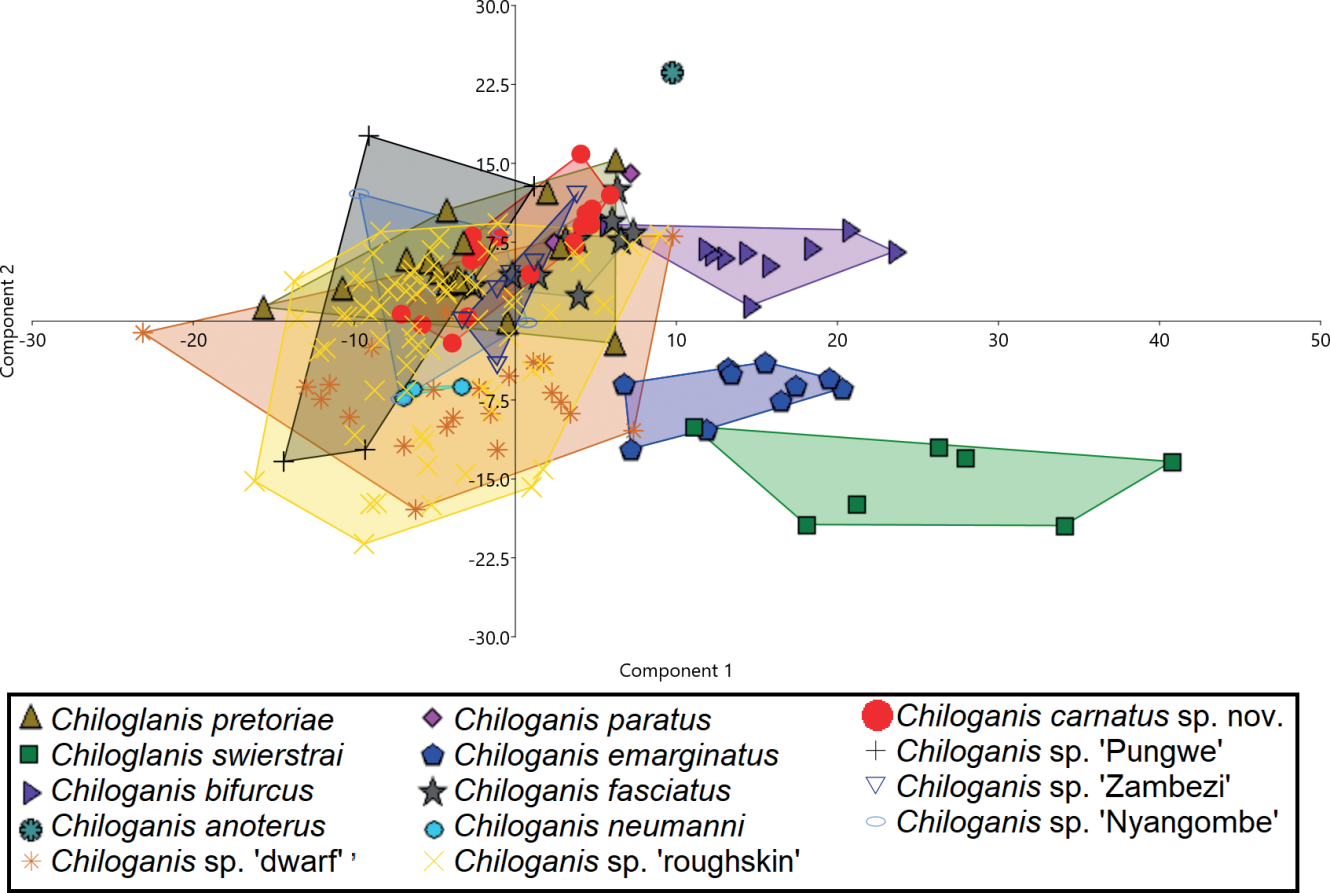
Scatter plot of the first two principal components of the morphometric characters of *Chiloglanis* species and lineages from southern African.

**Figure 7.**
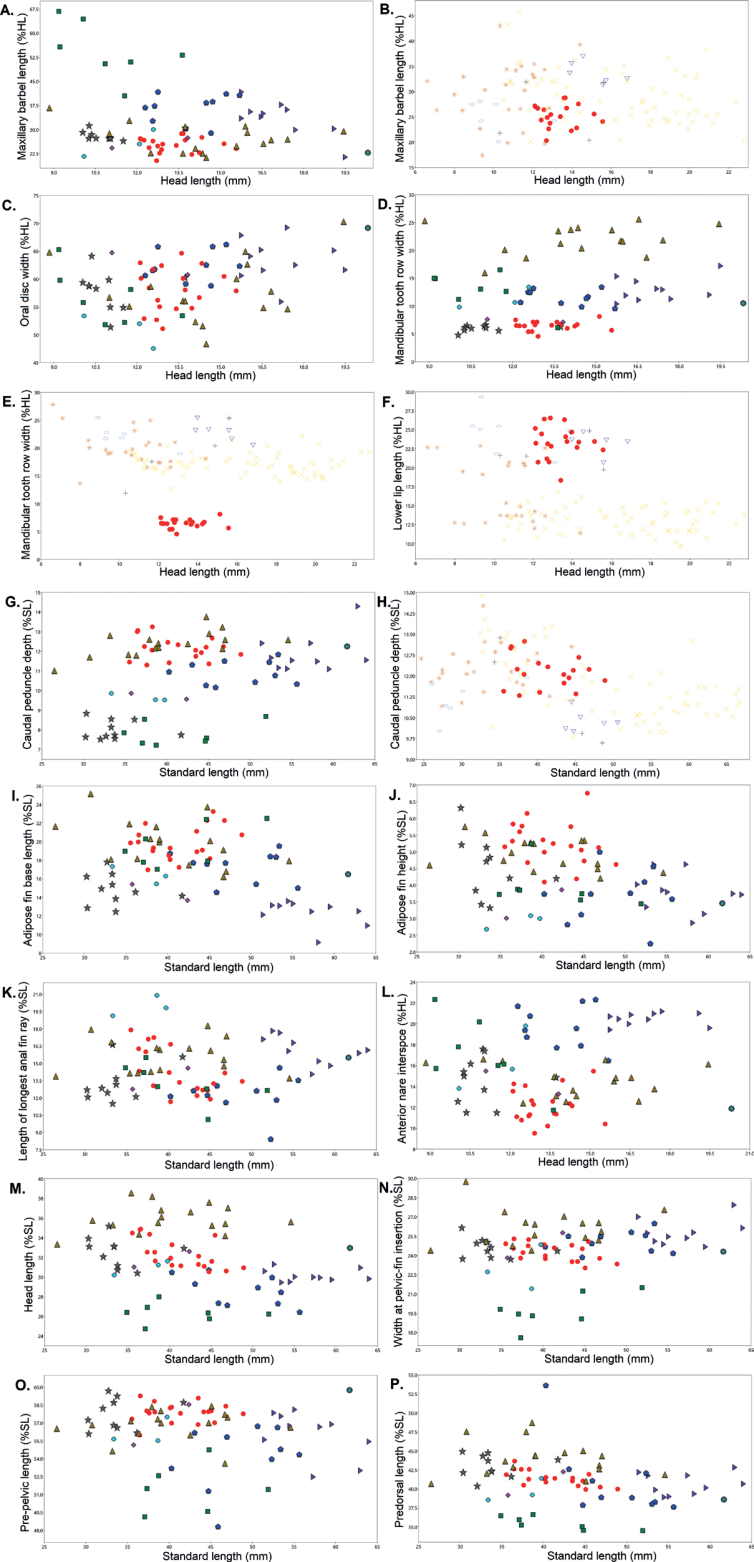
Scatterplots of the morphometric characters of the *Chiloglanis* species and lineages from southern African. Key: *Chiloglaniscarnatus* sp. nov. (red circle), *C.pretoriae* (brown triangle), *C.swierstrai* (dark green square), *C.bifurcus* (purple right-pointing triangle), *C.anoterus* (green heavy asterisk), *C.paratus* (pink diamond), *C.emarginatus* (Blue pentagon), *C.fasciatus* (grey star), *C.neumanni* (light blue circle), *Chiloglanis* sp. ‘dwarf’ (orange eight spoked asterisk), *Chiloglanis* sp. ‘roughskin’ (yellow multiplication sign), *Chiloglanis* sp. ‘Pungwe’ (black plus sign), *Chiloglanis* sp. ‘Zambezi’ (blue down-pointing hollow triangle), *Chiloglanis* sp. ‘Nyangombe’ (light blue hollow circle).

Principal component analysis (PCA) of the morphometric characters showed that *Chiloglaniscarnatus* sp. nov. is separated from *C.swierstrai* and *C.anoterus* along principal component 1 (PCI) (Fig. 6). This separation was associated with maxillary barbel length (Table 6). *Chiloglaniscarnatus* sp. nov. (20.3–28.8%HL) has relatively shorter maxillary barbels compared to *C.swierstrai* (44.2–66.8%HL) and *Chiloglanis* sp. ‘Zambezi’ (31.3–37.0%HL, Table 5, Fig. 7A, B). *Chiloglaniscarnatus* sp. nov. is separated from *C.swierstrai*, *C.anoterus*, and *C.neumanni* along principal component 2 (PCII) (Fig. 6). Separation along PCII is associated with the oral disc width (Table 6). *Chiloglaniscarnatus* sp. nov. has a relatively smaller oral disc width (51.1–64.6%HL) compared to *C.anoterus* (69.1%HL, Table 5, Fig. 7C).

**Table 5.** Summary of morphological characters examined in the present study. All values except standard length (SL) and Head length (HL) are given as percentages of the HL or SL. For the meristics the mode is given alongside the range of the counts in parentheses where the counts varied.

Species	*Chiloglaniscarnatus* sp. nov.	*Chiloglanispretoriae*	*Chiloglanisanoterus*	*Chiloglanisbifurcus*	*Chiloglanisemarginatus*	*Chiloglanisfasciatus*	*Chiloglanisneumanni*	*Chiloglanisparatus*	*Chiloglanisswierstrai*	*Chiloglanis* sp. ‘dwarf’	*Chiloglanis* sp. ‘Nyangombe’	*Chiloglanis* sp. ‘Pungwe’	*Chiloglanis* sp. ‘roughskin’	*Chiloglanis* sp. ‘Zambezi’
Number of specimens	19	16	1	10	10	10	3	2	7	25	6	4	65	6
Total length	45.3–62.2	31.7–67.1	80.1	68.7–84.9	50.2–66.6	37.7–53.3	0–42.7	44–51.9	45.2–65.7	31.4–51.1	33.1–48.2	34.4–62.9	39.8–87.6	55.2–62.6
Standard length	35.5–48.9	26.5–54.6	61.7	51.4–63.9	40.3–55.6	30.3–41.7	33.4–39.8	35.8–42.4	34.9–51.9	26.0–41.6	26.0–38.5	24.6–48.6	31.6–66.6	43.7–50.6
Head length	12.1–15.6	8.8–19.4	20.3	15.7–19.5	12.3–15.7	10.0–13.7	10.1–12.6	11.1–13.8	9.2–13.6	8.0–12.8	8.9–13.1	6.6–15.6	10.3–22.8	13.9–16.8
% Standard length														
Pre-pectoral length	26.9–30.0	26.3–32.7	30.9	27.4–31.1	24.5–27.3	29.5–32.4	26.1–27.8	28.4–30.0	25.0–27.0	24.3–33.1	29.6–32.3	30.2–35.9	25.7–32.4	25.8–31.4
Pre-dorsal length	39.9–43.7	40.7–48.7	38.6	38.9–42.9	37.7–42.7	40.4–44.9	38.6–41.5	39.2–42.3	34.5–36.7	36.6–50.5	39.0–46.8	35.4–44.7	36.0–44.1	36.2–46.3
Pre-pelvic length	56.0–59.3	53.6–58.4	59.7	52.5–58.1	51.3–56.8	56.1–59.7	55.6–57.5	55.2–58.6	49.1–54.8	50.7–58.6	51.7–64.2	57.2–65.1	53.6–61.6	55.3–60.7
Pre-anal length	67.6–73.3	66.4–72.8	73.3	63.4–69.3	63.0–68.2	67.7–73.3	70.7–72.4	67.6–71.3	64.1–69.5	64.1–75.3	65.2–80.1	56.7–80.2	64.3–77.6	58.7–76.5
Dorsal fin to adipose fin length	18.2–22.6	18.1–25.8	25.5	21.4–29.1	20.2–24.4	21.4–24.1	21.6–26.9	23.9–28	18.7–26.8	21.5–28.6	20.2–28.4	21.9–29.1	19.2–30.2	20.5–21.6
Pectoral-spine length	15.0–19.8	13.5–19.8	11.5	17.8–22.4	17.2–22	17.5–21.5	16.8–22.4	19.7–22.7	19.0–23.1	13.7–20	13.7–20.2	18.2–19.9	13.8–26.6	14.7–22.0
Pectoral-fin length	19.3–23.6	14.1–22.6	19	22.9–26.6	19.5–24.3	21.4–25.1	21.9–24.5	22.6–26.4	23.2–25.5	15.4–24.3	18.2–21.1	22.1–25.9	17.6–27.4	20.5–26.1
Width at pectoral-fin insertion	23.0–25.3	24.4–29.8	24.3	24.7–27.9	23.9–26.5	23.7–26.1	21.4–24.9	23.8–25.8	17.6–21.5	21.7–25.7	22.3–26.1	23.9–27.0	21.0–27.6	23.6–25.7
Pelvic-fin length	10.8–14.2	12.2–15.9	12.9	14.6–17.2	10.8–14.6	11.6–14.6	13.6–14	13.0–13.1	12.3–15.4	11.7–15.3	11.7–13.3	13.8–16.0	10.2–17.5	13.4–15.7
Pelvic-fin interspace	3.0–5.1	2.1–4.7	4.3	4.4–6.2	2.8–4.7	2.5–4.7	3.0–3.9	4.4–4.7	2.7–4.9	1.8–6.0	2.0–4.2	2.7–5.4	2.6–8.2	3.8–5.0
Body depth at dorsal-fin insertion	15.5–20.7	16.0–21.0	19.1	16.2–21.8	17.3–22.4	15.4–19.3	17.7–22.1	15.2–18.1	12.2–19.2	16.8–22.7	15.7–19.3	17.2–20.8	17.2–25.3	17.3–20.2
Body depth at anus	13.9–17.6	15.3–18.7	18	16.7–21.2	15.5–18.6	11.9–14.5	13.5–16.1	12.9–15.4	11.3–14.6	14.3–19.9	13.1–16.2	13.8–17.8	12.5–20.4	14.0–15.6
Dorsal-spine length	13.2–18.0	13.3–20.9	11.3	13–17.5	14.0–17.4	15.8–20.5	17.1–20.4	18.4–21.3	14.0–15.0	11.6–20.1	14.5–17.9	13.8–20.6	12.5–25.4	11.9–17.8
Dorsal-fin base length	10.7–14.1	12.8–18.0	8.6	9.5–13.2	8.8–12.9	10.4–13.7	8.5–10.2	12.8–14.6	7.5–9.6	11.5–23.7	12.8–16.6	20.6–28	11.8–30.6	15.4–24.7
Adipose fin to caudal peduncle length	12.9–17.0	13.3–17.7	15.2	14.9–18.5	13.1–17.0	13.1–16.8	11.9–14.1	13.6–15.6	14.0–15.9	14.4–19.8	15.1–20.9	11.3–16.1	13.3–21.6	15.7–17.2
Adipose-fin base length	17.0–23.3	16.2–25.2	16.5	9.2–13.6	14.6–19.6	12.4–17.8	15.5–17.4	13.7–15.4	17.0–22.5	13.2–17	11.3–17.3	13.2–19.6	10.4–19.3	14.9–17.4
Adipose-fin height	4.1–6.8	4.2–5.8	3.5	2.9–4.6	2.3–5.0	3.3–5.2	2.7–3.1	3.0–3.9	3.5–5.2	2.8–5.4	3.2–5.3	3.3–5.6	3.3–8.7	5.0–6.2
Anal-fin length along longest ray	11.7–17.9	13.7–18.3	15.5	14.1–17.8	11.6–14.6	11.5–16.6	19.2–20.9	12.8–14.6	10.1–15.5	11.8–18.7	13.0–17.2	11.0–17.5	10.9–20.5	13.2–16.9
Anal-fin base length	10.5–13.5	11.7–15.4	12.9	11.5–15.3	10.9–14.9	8.9–11.4	11.1–12.1	10.6–11.0	11.7–15	10.6–16.4	11.9–15.5	11.3–19.4	8.2–15.6	11.8–14.2
Caudal peduncle depth	11.3–13.2	11–13.8	12.2	11.1–14.1	10.2–11.9	7.5–8.8	9.5–9.9	9.6–9.9	7.2–8.7	10.9–12.6	10.0–11.9	9.6–12.8	9.8–14.9	10.0–11.1
Caudal peduncle length	15.9–19.7	15.8–22.6	18.6	19.9–22.7	19.1–23.7	18.8–21.7	16.0–18.8	17.7–18.9	19.6–22.0	17.4–24.5	20.3–22.9	13.6–15.8	13.9–21.9	16.2–17.9
Head length	30.5–34.9	33.3–38.6	32.9	29.5–31.3	26.5–30.7	30.4–35.2	30.2–31.7	31.0–32.6	24.8–28.0	27.8–34.9	32.8–39.4	25.9–33.7	28.3–36.3	31.4–34.1
% Head length														
Eye diameter (vertical axis)	9.9–13.8	11.6–18.3	10.6	12.1–16.6	11.9–16.5	9.4–13.1	9.1–14.9	10.6–12.5	13.2–18.6	11.8–16.1	12.6–13.9	10.9–20.4	7.4–15.3	11.5–15.1
Orbital interspace	21.5–28.7	22.6–28.9	23.4	19.5–24.6	18.3–24.4	18.5–25.4	25.7–30.2	22.2–23.9	15.3–22.7	23.3–38.5	20.9–25.2	23.8–38.4	18.0–38.9	22.7–30.4
Anterior nares interspace	9.5–15.5	12.4–16.6	11.9	19.5–21.2	16.5–22.4	11.5–17.6	13.9–19.8	13.3–15.5	15.7–22.4	10.9–18.4	13.0–14.7	13.7–23.0	11.0–21.6	13.5–16.7
Posterior nares interspace	10.3–15.5	11–15.5	11.8	15.3–21.9	13.8–20.9	9.9–16.1	14.9–22.2	9.3–10.9	12.6–18.2	12.9–16.7	12.6–14.7	8.9–13.0	7.5–18.4	10.2–13.0
Snout length	54.0–66.2	55.7–65.7	65	58.2–64.8	49.5–59.9	58.9–69.3	51.5–56.2	56.9–59.0	51.5–57.5	52.3–66.1	51.4–67.3	54.7–84	51.1–68.0	53.4–66.2
Pre-maxillary tooth-patch length	8.2–12.3	8.2–13.9	11.7	7.0–9.3	6.3–8.2	7.5–9.5	9.9–12.9	9.2–11.8	8.5–11.6	6.8–10.3	6.1–9.9	7.5–11.5	5.4–14.3	9.9–12.1
Pre-maxillary tooth-patch width	36.8–47.9	30.7–46.8	51.5	44.1–50.2	39.9–46.2	40.3–46.7	36.4–38.9	41.3–44.2	39.5–47.1	35.9–45.3	39.6–47.8	31.7–44.5	29.7–50.1	37.2–45.1
Mandibular tooth row width	4.6–8.1	16.0–25.6	10.5	10.4–17.3	9.6–13.5	4.8–6.6	9.9–13.5	7.2–7.7	10.0–16.6	13.6–25.0	19.0–25.5	17.6–27.8	11.9–22.2	20.5–25.4
Maxillary barbel length	20.3–28.8	21.3–36.8	22.7	23.8–41.8	29.1–41.8	26.4–31.2	21.8–30.2	24.3–27.5	44.2–66.8	17.4–34.3	20.1–28.1	19.7–32.1	20.2–45.7	31.3–37.0
Upper lip length	11.1–16.2	11.7–18.8	16.7	8.4–12.3	6.6–10.6	8.8–15.5	9.1–10.0	12.3–12.4	7.0–10.5	6.9–14.3	8.4–13.5	11.3–19.5	10.2–19.5	12.5–17.2
Lower lip length	18.3–26.6	22.4–27.7	25.1	17.7–25.6	23.5–28.8	18.8–24.9	19.8–27.0	22.8–27.6	19.2–26.0	12.6–22.9	20.7–29.3	19.7–27.8	9.6–16.8	20.7–24.7
Mouth width	23.9–33.8	24.8–32.6	39.6	30.9–39.1	25.7–36.2	25.6–32.1	20.6–22.8	28.0–35.9	27.9–34.0	20.4–33.4	27.1–33.8	25.2–37.8	17.5–32.8	24.7–31.1
Oral disc width	51.1–64.6	48.4–70.3	69.1	59.3–69.3	58.9–66.3	51.4–64.1	47.6–53.5	60.8–64.8	51.8–63.0	44.4–57.3	45.8–56.1	63.4–69.7	35.7–69.2	54.4–63.3
Oral disc length	48.6–57	46.0–61.1	63.8	53.7–61.3	43.3–54.7	48.4–59.7	41.6–53.2	55.7–57.8	48.6–62.4	40.7–53.9	41.9–58.2	46.5–57.4	38.6–57.1	47.9–57.3
Meristics														
Mandibular tooth count	10	12	12	8	8 (6–8)	8	8	12	11 (11–14)	_	_	_	_	_
Pre-maxillary tooth count	60 (43–69)	51–59	86	54 (50–64)	36–54	64 (51–65)	55–60	39–51	50 (34–59)	_	_	_	_	_
Pectoral-fin ray count	8 (6–8)	8	8	8 (7–8)	7	8	8	8	8	_	_	_	_	_
Pelvic-fin ray count	7 (6–7)	7	7	7	7 (7–8)	7	7	7	7	_	_	_	_	_
Dorsal-fin ray count	6 (5–7)	6	5	5 (5–6)	6 (5–6)	6 (5–6)	5	5	5 (5–6)	_	_	_	_	_
Anal-fin ray count	12 (12–13)	10	13	12 (9–13)	10 (10–12)	9 (8–12)	10 (10–12)	9	12 (9–13)	_	_	_	_	_
X-rays														
Number of specimens	9	2	1	4	6	7	5	1	6	_	_	_	_	_
Abdominal vertebrae	13 (11–13)	12–13	13	11 (10–11)	10 (10–12)	13 (12–13)	13 (12–13)	12	12 (11–13)	_	_	_	_	_
Caudal vertebrae	17 (16–18)	18–19	17	20 (18–20)	19 (17–19)	16 (16–17)	16 (16–18)	16	20 (16–20)	_	_	_	_	_
Total vertebrae	29 (29–30)	28	30	30 (29–30)	29 (28–30)	29 (28–29)	28	27	32 (31–32)	_	_	_	_	_

**Table 6.** The PCA loadings for the first two principal components for the measured characters of *Chiloglanis* species and lineages from southern Africa.

Principal component	1	2
Eigenvalue	90.91	70.84
% variance	22.46	17.50
Adipose fin to caudal peduncle length	-0.02	-0.02
Adipose-fin base length	0.05	-0.01
Adipose-fin height	-0.04	0.00
Anal-fin base length	0.03	-0.05
Anal-fin length along longest ray	-0.03	-0.06
Body depth at anus	-0.02	-0.04
Body depth at dorsal-fin insertion	-0.09	-0.06
Caudal peduncle depth	-0.05	-0.02
Caudal peduncle length	0.08	-0.10
Dorsal-fin to adipose fin length	-0.02	-0.02
Dorsal-fin base length	-0.17	0.08
Dorsal-spine length	-0.06	0.04
Pre-anal length	-0.16	0.17
Pre-dorsal length	-0.10	0.11
Pre-pectoral length	-0.08	0.10
Pre-pelvic length	-0.12	0.15
Pectoral-spine length	0.04	0.01
Pectoral-fin length	0.04	0.03
Pelvic-fin length	0.00	0.02
Width at pectoral-fin insertion	-0.04	0.06
Pelvic-fin interspace	0.00	0.02
Head length	-0.18	0.07
Anterior nares interspace	0.14	-0.08
Eye diameter (vertical axis)	0.11	-0.03
Lower lip length	0.02	0.37
Mandibular tooth row width	-0.16	-0.05
Maxillary barbel length	**0.60**	-0.41
Mouth width	0.35	0.21
Orbital interspace	-0.18	-0.14
Oral disc length	0.22	0.34
Oral disc width	0.39	**0.41**
Pre-maxillary tooth-patch length	0.00	0.09
Pre-maxillary tooth-patch width	0.23	0.24
Posterior nares interspace	0.13	-0.13
Snout length	-0.05	0.35
Upper lip length	-0.09	0.15

**Table 7.** Summary of morphological characters for *Chiloglaniscarnatus* sp. nov. All values except standard length (SL) and Head length (HL) are given as percentages of the HL or SL.

	Holotype	Paratypes			
	Male	Males		Females	
Number of specimens		7		11	
		Range	Mean	Range	Mean
Total length	58.2	45.3–62.2	49.8	45.3–56.1	52.2
Standard length	46.8	36.5–48.9	39.6	35.5–45.5	41.8
Head length	14.3	12.1–15.1	13.0	12.3–15.6	13.5
% Standard length					
Pre-pectoral length	28.1	26.9–30.0	28.9	27.1–29.1	28.3
Pre-dorsal length	40.2	40.0–42.6	41.6	39.9–43.7	41.3
Pre-pelvic length	58.4	56.0–58.8	57.8	56.9–59.3	57.9
Pre-anal length	71.1	67.6–70.8	69.1	67.9–73.3	70.6
Dorsal fin to adipose fin length	20.9	18.4–22.2	20.6	18.2–22.6	20.6
Pectoral-spine length	18.6	15.6–19.8	17.7	15.0–18.6	16.5
Pectoral-fin length	20.9	20.9–23.6	22.4	19.3–22.2	20.9
Width at pectoral-fin insertion	23.8	23.3–25.2	24.3	23.0–25.3	24.3
Pelvic-fin length	12.2	13.3–14.2	13.7	10.8–14.1	12.3
Pelvic-fin interspace	4.6	3.3–4.6	4.0	3.0–5.1	3.9
Body depth at dorsal-fin insertion	18.9	15.5–20.7	18.0	16.2–20.1	17.8
Body depth at anus	17.6	15.3–16.9	15.8	13.9–17.0	15.9
Dorsal-spine length	15.7	13.6–18.0	16.1	13.2–17.7	15.9
Dorsal-fin length along longest ray	17.9	15.2–20.7	18.5	16.2–20.0	17.4
Dorsal-fin base length	11.0	12.1–14.1	13.1	10.7–13.8	12.3
Adipose fin to caudal peduncle length	13.5	12.9–17.0	15.0	10.3–16.4	13.8
Adipose-fin base length	22.3	17.0–22.0	19.6	17.2–23.3	19.8
Adipose-fin height	5.1	4.1–6.1	5.2	4.2–6.8	5.3
Anal-fin length along longest ray	14.2	13.1–17.2	15.7	11.7–17.9	13.4
Anal-fin base length	12.1	11.8–15.3	13.2	11.1–13.4	12.5
Caudal peduncle depth	12.2	11.3–13.2	12.1	11.4–13.1	12.1
Caudal peduncle length	16.8	16.0–19.2	18.3	15.9–19.7	17.5
Caudal fork length	12.3	9.8–14.5	11.4	9.2–14.4	11.7
Head length	30.6	30.9–34.8	32.9	30.5–34.9	32.2
% Head length					
Head depth	57.4	43.9–57.6	51.2	48.2–57.3	51.2
Eye diameter (vertical axis)	11.9	10.6–13.2	11.7	9.9–13.8	11.9
Eye diameter (horizontal axis)	15.7	13.0–16.4	14.1	12.9–16.8	15.0
Orbital interspace	25.1	22.3–28.7	24.1	21.5–26.8	24.5
Anterior nares interspace	12.1	9.5–15.5	12.1	10.4–14.6	12.2
Posterior nares interspace	12.6	11.0–15.5	13.5	10.3–15.4	12.7
Snout length	61.1	54.3–63.8	58.7	54.0–66.2	60.7
Pre-maxillary tooth-patch length	9.9	8.2–11.0	9.9	8.8–12.3	10.4
Pre-maxillary tooth-patch width	44.3	36.8–44.7	41.1	38.4–47.9	42.1
Mandibular tooth row width	6.7	4.6–8.1	6.4	5.4–7.1	6.4
Maxillary barbel length	27.6	20.3–27.2	25	22.3–28.8	25.3
Upper lip length	15.1	11.1–14.5	13.1	11.3–16.2	13.9
Lower lip length	23.4	18.3–25.2	22.7	20.7–26.6	23.8
Mouth width	29.2	25.3–30.8	27.3	23.9–33.8	28.1
Oral disc width	62.8	51.1–62.9	57.2	52.9–64.6	58.2
Oral disc length	54.3	48.6–57.0	53.1	50.2–56.4	53.3
Postcleithral process to occipital shield	37.8	29.5–36.3	33.1	32.2–38.3	35.5
Length of postcleithral process	29.6	23.4–28.9	25.5	22.9–27.8	25.9
Occipital shield width	23.6	14.6–19.5	16.9	14.9–24.2	18.8
Lower caudal-fin lobe length	13.4	9.3–13.0	10.6	10.0–12.7	11.2
Upper caudal-fin lobe length	10.8	8.7–12.1	9.8	9.1–11.7	10.4
Medial mandibular barbel length	0.6	0.2–0.6	0.4	0.4–0.9	0.6
Lateral mandibular barbel length	1.3	1.0–1.8	1.4	1.1–1.8	1.4
Meristics		**Range**	**Mode**	**Range**	**Mode**
Mandibular tooth count	10	8–10	10	8–10	10
Pre-maxillary tooth count	59	43–69	_	49–68	60
Pectoral fin-ray count	8	7–8	8	6–8	8
Pelvic fin-ray count	7	7	7	6–7	7
Dorsal fin-ray count	6	6	6	5–7	6
Anal fin-ray count	13	12–13	12	12–13	12
Abdominal vertebrae	12	12	_	11–13	13
Caudal vertebrae	17	17	_	16–18	16
Total vertebrae	29	29	_	29–30	29

Additional scatterplots were generated to explore the characters that further distinguish the *Chiloglaniscarnatus* sp. nov. specimens. The *Chiloglaniscarnatus* sp. nov. specimens have a narrower mandibular tooth row width (4.6–8.1%HL) compared to *C.pretoriae* (16.0–25.6%HL), *C.swierstrai* (10.0–16.6%HL), *C.neumanni* (9.9–13.5%HL), *C.emarginatus* (9.6–13.5%HL), *C.bifurcus* (10.4–17.3%HL), *C.anoterus* (10.5%HL), *Chiloglanis* sp. ‘dwarf’ (13.6–25.0%HL), *Chiloglanis* sp. ‘Nyangombe’ (19.0–25.5%HL), *Chiloglanis* sp. ‘Pungwe’ (17.6–27.8%HL), *Chiloglanis* sp. ‘roughskin’ (11.9–22.2%HL), and *Chiloglanis* sp. ‘Zambezi’ (20.5–25.4%HL; Fig. 7D, E). *Chiloglaniscarnatus* sp. nov. has an oral disc with relatively longer lower lips (18.3–26.6%HL) compared to *Chiloglanis* sp. ‘roughskin’ (9.6–16.8%HL, Fig. 7F). *Chiloglaniscarnatus* sp. nov. has a relatively deeper caudal peduncle (11.3–13.2%SL) compared to *C.neumanni* (9.5–9.9%SL), *C.paratus* (9.6–9.9%SL), *C.fasciatus* (7.5–8.8%SL), *C.swierstrai* (7.2–8.7%SL), and *Chiloglanis* sp. ‘Zambezi’ (10.0–11.1%SL, Fig. 7G, H). A longer adipose-fin base length distinguishes *Chiloglaniscarnatus* sp. nov. (17.0–23.3%SL) from *C.bifurcus* 9.2–13.6%SL) (Fig. 7I). Larger adipose fin height (4.1–6.8%SL) and shorter anal fin rays (11.7–17.9%SL) further distinguish *Chiloglaniscarnatus* sp. nov. from *C.neumanni* (adipose fin height: 2.7–3.1%SL; anal fin ray length: 19.2–20.9%SL; Fig. 7J, K). A shorter distance between the anterior nares of *Chiloglaniscarnatus* sp. nov. (9.5–15.5%HL) separates it from *C.bifurcus* (19.5–21.2%HL), *C.emarginatus* (16.5–22.4%HL), and *C.swierstrai* (15.7–22.4%HL, Fig. 7L). *Chiloglaniscarnatus* sp. nov. has a relatively longer head (30.5–34.9%SL vs 24.8–28.0%SL), relatively wider body at pectoral-fin insertion (23.0–25.3%SL vs 17.6–21.5%SL), and relatively longer pre-pelvic (56.0–59.3%SL vs 49.1–54.8%SL) and pre-dorsal distances (39.9–43.7%SL vs 34.5–36.7%SL) that readily separated them from *C.swierstrai* (Fig. 7M–P).

**Figure 8.**
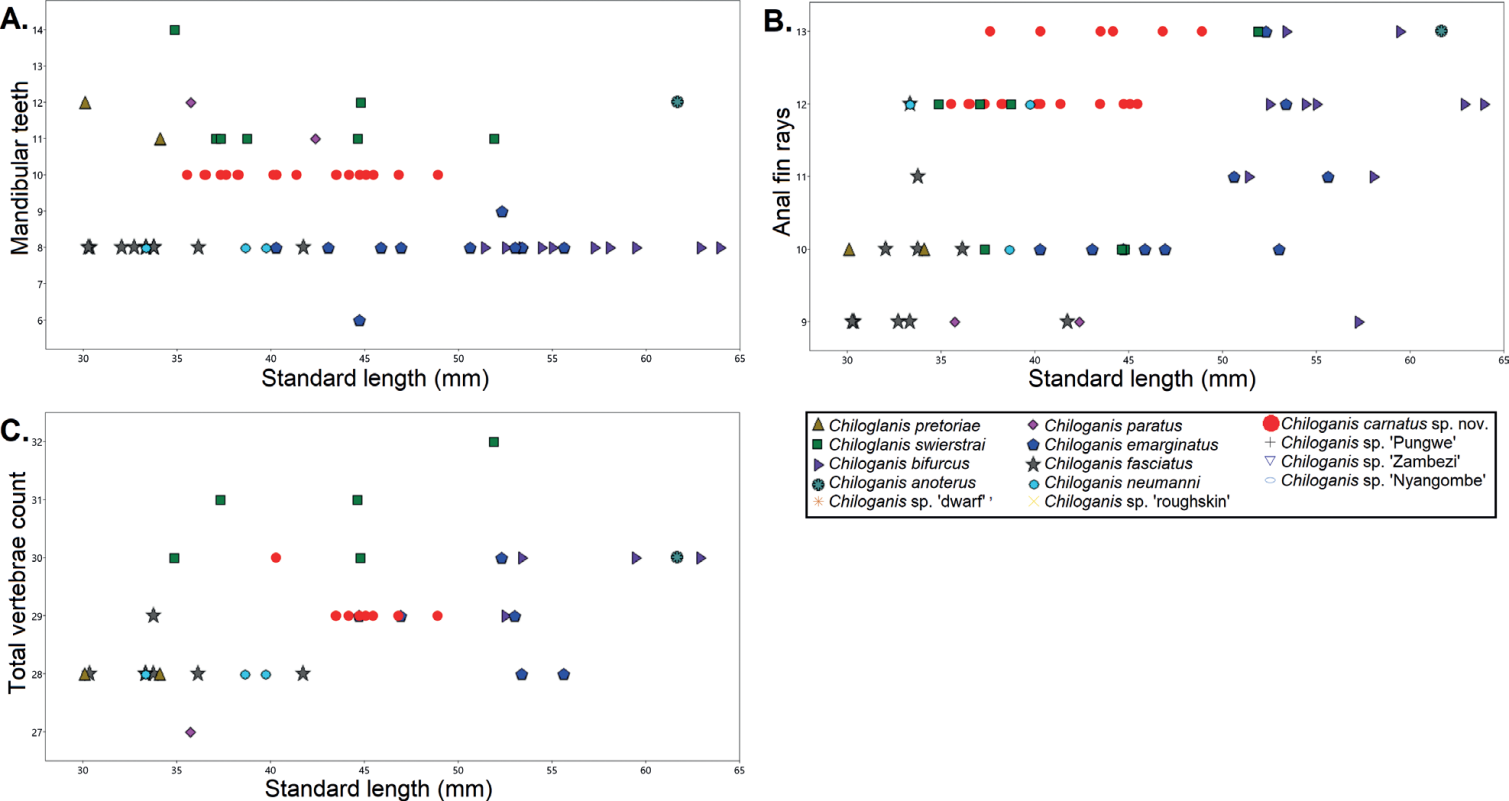
Scatterplots of the meristic characters of the *Chiloglanis* species from southern African.

Comparison of meristic characters revealed consistent differences between *Chiloglaniscarnatus* sp. nov. specimens and the other species from southern Africa. *Chiloglaniscarnatus* sp. nov. specimens have ten closely packed mandibular teeth that separate them from *C.bifurcus*, *C.emarginatus*, *C.fasciatus*, and *C.neumanni* that have eight mandibular teeth as well as from *C.anoterus*, *C.pretoriae*, *C.paratus*, and *C.swierstrai* that have > 10 mandibular teeth (Fig. 8A). A higher number of anal-fin rays separates *Chiloglaniscarnatus* sp. nov. specimens (12–13) from *C.paratus* (9) and *C.pretoriae* (10) (Fig. 8B). *Chiloglaniscarnatus* sp. nov. specimens have a higher number of total vertebrae (29–30) compared to *C.neumanni* (28), *C.pretoriae* (28), and *C.paratus* (27) (Fig. 8C).

The integrated approach used in this study provided genetic and morphological characters that clearly and consistently distinguish *Chiloglaniscarnatus* sp. nov. from the known species and lineages from this region. This study has thus provided evidence that supports the description of the *Chiloglaniscarnatus* sp. nov. as a new species.

### ﻿Taxonomic account

#### ﻿ Chiloglanis carnatus

Taxon classificationAnimalia

Mutizwa, Bragança & Chakona sp. nov.

8CFDB994-A475-5916-8EA9-F0D21DE7495F

https://zoobank.org/E1F0912C-986F-450F-9B90-400D86F5F3BC

##### Material examined.

**Figure 9.**
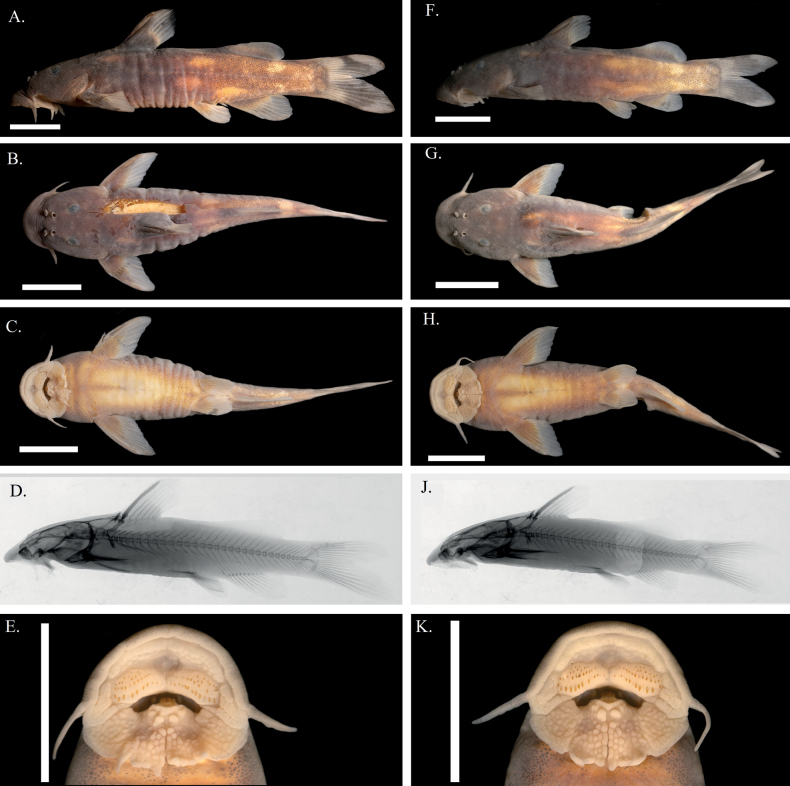
Holotype of *Chiloglaniscarnatus* sp. nov., SAIAB 236631 male (**A–E**) and female paratype specimen SAIAB 211346 (**F–K**). Scale bars: 1 cm.

***Holotype*.** Zimbabwe • ♂, stored in 70% ethanol, 46.8 mm SL, Fig. 9A–E; Mukwadzi River near bridge on the road to Mutorashanga, Manyame River sub-catchment, middle Zambezi River system, Mashonaland West Province, 17.42485°S, 30.58542°E; 30 Jun. 2016; A. Chakona, W. Kadye and T. Bere; SAIAB 236631; genseq-1 COIPP156890. ***Paratypes*.** Zimbabwe • 5 ♀, stored in 70% ethanol, 36.5–45.5 mm SL; near bridge on the road to Mutorashanga, Mukwadzi River, Manyame River sub-catchment, middle Zambezi River system, Mashonaland West Province, 17.42485°S, 30.58542°E; 30 Jun. 2016; A. Chakona, W. Kadye and T. Bere; SAIAB 211346; genseq-2 COIPP156891 to PP156895. Zimbabwe • 6 ♀, 35.5–45.1 mm SL, 7 ♂, 36.5–48.9 mm SL, stored in 70% ethanol; near bridge on the road to Mutorashanga, Mukwadzi River, Manyame River system, middle Zambezi Basin, Mashonaland West Province, 17.42444°S, 30.58453°E; 11 Apr. 2019; A. Chakona, W. Kadye and T. Bere; SAIAB 211349.

##### Diagnosis.

*Chiloglaniscarnatus* sp. nov. is readily distinguished from its congeners in southern Africa (i.e. *C.anoterus*, *C.bifurcus*, *C.emarginatus*, *C.fasciatus*, *C.paratus*, *C.pretoriae* and *C.swierstrai*) by the presence of a dorsal fin that has a basal portion covered by a fleshy skin, a character which is absent in the other species. *Chiloglaniscarnatus* possesses ten closely packed mandibular teeth, that further distinguishes it from *C.fasciatus* that has eight closely packed mandibular teeth; *C.bifurcus* and *C.emarginatus* that have eight widely spaced mandibular teeth; *C.anoterus*, *C.paratus*, and *C.pretoriae* that have 12 closely packed mandibular teeth; and *C.swierstrai* that has 14 closely packed mandibular teeth. *Chiloglaniscarnatus* possesses a deeply forked caudal fin that readily separates it from *C.pretoriae* and *C.emarginatus* that have emarginate caudal fins, and from *C.anoterus* that has a caudal fin with extended median rays in males and emarginate in females. *Chiloglaniscarnatus* possesses a caudal fin with an upper lobe that is shorter than the lower lobe. This distinguishes it from *C.bifurcus* that has a caudal fin with an upper lobe that is longer than the lower lobe. *Chiloglaniscarnatus* has an oral disc with a well-developed mid-ventral cleft that distinguishes it from *C.swierstrai* that possesses an oral disc without a mid-ventral cleft. *Chiloglaniscarnatus* possesses a smooth skin with a few tubercles occasionally found on the head that separates it from *C.fasciatus* that has its entire dorsal and lateral body surfaces mostly covered by small tubercles. *Chiloglaniscarnatus* has a dorsal spine with crenate anterior and posterior margins that distinguish it from *C.paratus* that has a dorsal spine with a serrated posterior margin.

##### Description.

Morphometric proportions and meristics are summarised in Table 7. Holotype meristic counts are given in parentheses.

***Body shape*.** Anterior portion of body slightly compressed dorsally, becoming laterally compressed from pelvic fin insertion to caudal peduncle. Body greatest depth at dorsal-fin insertion. Pre-dorsal profile convex, sharply slopping from snout to posterior nostril, gently from nostril to dorsal-fin origin. Post-dorsal profile about straight from dorsal-fin base to adipose-fin origin, becoming gently concave from adipose-fin origin to caudal fin. Ventral profile gently convex from region just posterior to oral disc to anal-fin origin, becoming gently concave from anal-fin origin to caudal fin.

***Head*.** slightly depressed dorsally. Oval eye dorsally positioned, ~ 1/2 distance between snout and gill opening. Interorbital distance greater than distance between nostrils. Anterior and posterior nostrils closer to the eye than snout. Distance between anterior nostrils slightly greater than distance between posterior nostrils. Posterior nostril medially positioned relative to orbit. Anterior nostril with posterior flap; posterior nostril with anterior flap. Occipital-nuchal shield not visible through skin. Gill opening above pectoral fin insertion.

***Oral disc*.** Mouth inferior; large upper and lower lips combined to form oral disc (see Fig. 9E, K). Oral disc width greater than length. Upper and lower lips with pronounced roundish papillae, largest papillae concentrated around mid-ventral cleft of lower lip. Three pairs of barbels. Maxillary barbel unbranched, originating from lateral region of oral disc, extending to posterior region of oral disc. Lateral mandibular barbel longer than medial mandibular barbel, both incorporated into lower lip. Shallow cavity above lower lip.

***Dentation*.** Pre-maxillary teeth arranged in three or four rows; variable number of teeth (43–69). Up to 5+5 closely packed mandibular teeth; central teeth projecting higher than outer teeth forming a gentle arc; replacement tooth row emerges anteriorly to the functional row.

**Figure 10.**
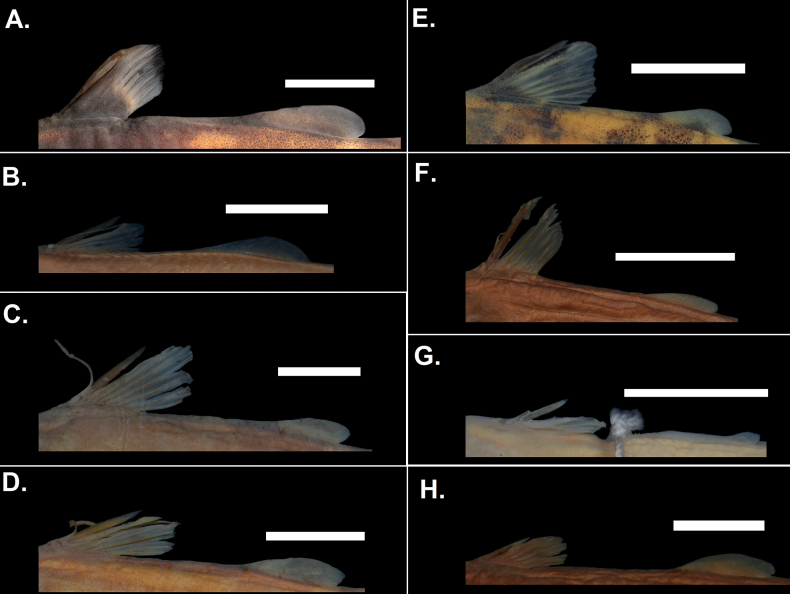
Comparison of the dorsal and adipose fins of *Chiloglaniscarnatus* sp. nov. and the type specimens of the valid southern African species **A***Chiloglaniscarnatus* sp. nov. (SAIAB 236631) specimens have an extended dermal tissue covering the base of the dorsal fin that distinguishes them from **B***C.swierstrai* (SAIAB 186247) **C***C.bifurcus* (SAIAB 120160) **D***C.emarginatus* (SAIAB 120117) **E***C.fasciatus* (SAIAB 204928) **F***C.paratus* (SAIAB 186248) **G***C.pretoriae* (SAIAB 30011) **H***C.anoterus* (SAIAB 186246). Scale bars: 1 cm.

***Fins*.** Dorsal-fin ray count 5–7 (6), originating in anterior 1/3 of body, posterior to pectoral-fin origin. Dorsal fin basal portion covered by a fleshy skin prominent in large adult males and females with ~ ¾ of the dorsal spine and the first two rays also covered by fleshy skin (Fig. 10). Dorsal spine length ~ 80% of longest dorsal fin ray length. Dorsal spine with dentate anterior and posterior margins. Pectoral-fin ray count 6–8 (8), origin anterior to gill opening; pectoral spine anterior margin smooth; dentate posterior margin; pectoral spine length ~ 80% of pectoral fin length. Adipose fin origin preceded by anterior tissue flange; rounded (Fig. 10). Caudal fin forked, lower lobe longer than upper lobe. Anal-fin ray count 12 or 13 (13), origin posterior to origin of adipose fin; terminating just before end of adipose-fin; rounded. Pelvic-fin ray count 6 or 7 (7), origin posterior to midpoint between end of dorsal-fin and adipose-fin origin; rounded.

***Skin*.** Skin smooth with occasional tubercles present, concentrated on dorsal and lateral surface of head. Lateral line complete; originating anterior to dorsal fin at same horizontal level of orbit and sloping ventrally until it lies mid-laterally along body.

***Sexual dimorphism*.** Urogenital opening situated adjacent to origin of anal fin. Urogenital papillae sexually dimorphic; elongated in males; reduced and separated from anus by shallow invagination in females.

***Colouration*.** Overall body background colouration brown with yellowish ventral surface. Anterior portion of body dark brown becoming paler towards posterior. Small dark melanophores scattered across entire dorsal and lateral sides. Six yellowish brown blotches on lateral surface of body; two vertically arranged posterior to end of adipose fin; one above origin of anal fin; two above pelvic fin origin; and one below dorsal fin origin. Basal 1/3 of fins pale to dark brown with medium and posterior portion of fins gradually becoming translucent. Dark blotch cuts vertically across caudal peduncle lobes.

***Vertebral counts*.** Total vertebrae 29 or 30 (29), abdominal vertebrae 11–13 (12), caudal vertebrae 16–18 (17).

##### Etymology.

The specific epithet *carnatus* means fleshy, referring to the dermal tissue covering the base of the dorsal fin of some of the larger specimens of this species and the general robust body structure of this species compared to its regional congeners.

##### Distribution.

*Chiloglaniscarnatus* was collected from two sites in the Mukwadzi River near the bridge on the Mutorashanga Road. The Mukwadzi River is a perennial river that originates from wetlands (dambos) on the eastern side of the Great Dyke. This river flows in a north-western direction cutting through the Great Dyke before it joins the Manyame River. The Great Dyke is a major intrusion of mafic and ultramafic rocks that have vast ore deposits, including gold, silver, chromium, platinum, nickel, and asbestos. The rich mineral deposits have resulted in the establishment of many mines along the Great Dyke. The sites where *C.carnatus* was collected were in a communal area surrounded by rural communities on the western slope of the Great Dyke. The substratum at the sites was composed of bedrock, cobbles and gravel, and the riparian vegetation was dominated by *Syzygium* Gaertner, 1788 and *Phragmites* Adanson, 1763. At these sites *C.carnatus* co-occurred with native fish species that include *Labeocylindricus* Peters, 1852, *Opsaridiumzambezense* (Peters, 1852), *Enteromiustrimaculatus* (Peters, 1852), *Tilapiasparrmanii* Smith, 1840, *Clariasgariepinus* (Burchell, 1822), and *Labeobarbusmarequensis* (Smith, 1841) as well as the non-native species *Serranochromisjallae* (Boulenger, 1896) and *Micropterussalmoides* (Lacepède, 1802).

## ﻿Discussion

This study integrated molecular and morphological data to evaluate the taxonomic distinctiveness of specimens of suckermouth catfishes that were collected from the middle Zambezi River system in Zimbabwe. Based on substantial genetic differentiation as well as consistent meristic, morphometric, and qualitative differences from its southern African congers, a new species of *Chiloglanis* is described. This is the first description more than five decades after the last comprehensive review of *Chiloglanis* species from southern Africa (see Jubb and Le Roux 1969). This study adds to the growing body of literature that demonstrates the value of integrative taxonomic approaches in the discovery and description of new species within this region (Maake et al. 2014; Morris et al. 2016; Riddin et al. 2016; Kambikambi et al. 2021; Mazungula and Chakona 2021). As evidenced from this study and work by Chakona et al. (2018), additional species of suckermouth catfishes from southern Africa remain to be formally described. It is anticipated that ongoing taxonomic studies on this group of fishes will result in the description of at least ten new species from this region. These species were all previously included under a single species, *C.neumanni*, but this study and ongoing work by researchers from the NRF-SAIAB indicates that this species does not occur in southern Africa. Updated taxonomic information of *Chiloglanis* species from this region will improve our understanding of biogeographic and phylogeographic patterns as well as drainage evolution in the region.

The dentition of species in the genus *Chiloglanis*, like that of most members of the family Mochokidae, is highly specialised (Roberts 1989). *Chiloglaniscarnatus* possesses ten closely packed mandibular teeth, a number not found in any other *Chiloglanis* species in southern Africa. Variation in the number of mandibular teeth in individual specimens can be observed due to tooth loss from the functional row, delayed exposure of some teeth in the replacement row, or early advancement of some replacement row teeth (Roberts 1989). However, by examining both the functional and replacement rows, it was possible to determine the diagnostic number of teeth for this species. Outside southern Africa, the presence of ten mandibular teeth has been reported in west African species such as *C.kolente*Schmidt et al., 2017, *C.kabaensis*Schmidt et al., 2017, *C.nzerekore*Schmidt et al., 2017, *C.occidentalis* Pellegrin, 1933, and *C.normani* Pellegrin, 1933 (Paugy et al. 2003; Schmidt et al. 2017). In addition to dentation, there were several morphometric characters associated with the oral disc (e.g., maxillary barbel length, oral disc width, lower lip length and mandibular tooth row width) that distinguish *C.carnatus* from congenerics in southern Africa. Considering the importance of the oral disc in the ecology of the species in this genus, these differences warrant further study, particularly assessing potential differences in trophic ecology.

Rheophilic habitats form ‘islands’ with suitable environmental conditions for specialised taxa such as those in the genus *Chiloglanis*. The disjunct distribution of these habitats within a river may play an important role in promoting genetic and morphological diversity within rheophilic taxa. Some rheophilic species have very narrow distribution ranges such that significant differences have been found in the fish communities occurring at different rapids within the same river system (Hrbek et al. 2018). In southern Africa high genetic and morphological diversity within *C.anoterus* has been reported from geographically isolated populations in the upper sections of the Phongolo and Inkomati river systems, highlighting the importance of the rheophilic habitats in headwater streams (Morris et al. 2016). The close association of *Chiloglanis* species with rheophilic habitats probably promotes diversification; however, this has yet to be explicitly tested within this region. The discovery of the *C.carnatus* from a small section of the Mukwadzi River as well as other undescribed species within southern Africa (Chakona et al. 2018) emphasises the need for accelerating inventory of the diversity found in rheophilic habitats as these may harbour a considerable number of species which are still unknown to science.

A number of southern African freshwater fish species in the genera *Enteromius* Cope, 1867, *Nothobranchius* Peters, 1868, *Pseudobarbus* Smith, 1841, *Sandelia* Castelnau, 1861, *Galaxias* Cuvier, 1816, and *Oreochromis* Günther, 1889 are threatened with extinction due to their narrow geographic ranges, the introduction of invasive species, and habitat degradation (Marshall and Tweddle 2007; Jordaan and Chakona 2017; Roux and Hoffman 2017b; Nagy and Watters 2019). Among the *Chiloglanis* species from southern Africa, *C.bifurcus* and *C.emarginatus* are under threat with the former classified as Critically Endangered and the latter as Vulnerable in the IUCN Red List of threatened species (Roux and Hoffman 2017a, 2018). *Chiloglanisbifurcus* is a narrow-range endemic species that is confined to the upper sections of the Inkomati River system, whereas *C.emarginatus*’ range in the Phongolo River system has declined substantially over the past decades (Roux and Hoffman 2017a, 2018). Habitat loss though flow regulation, pollution, and sedimentation has been attributed as the main driver of population decline in both these species (Roux and Hoffman 2017a, 2018). *Chiloglaniscarnatus* was collected from two sites in the Mukwadzi River. The section downstream of these sites as well as other tributaries of the Mukwadzi River are heavily impacted by anthropogenic activities. There are at least 13 small impoundments in the Mukwadzi River before its confluence with the Manyame River. Largemouth bass (*Micropterussalmoides*) and the nembwe (*Serranochromisjallae*) were also introduced into this river system, and this combination of flow modification, water abstraction, and non-native species is likely to negatively impact populations of native species (Gratwicke and Marshall 2001; Gratwicke et al. 2003; Kadye et al. 2013; Kadye and Booth 2020). In addition to the modification of this river and the non-native species, the rich mineral resources found within the Great Dyke attract formal and informal mining operations which also threaten the species living within these rivers through increased sedimentation/siltation which may cause habitat loss. Although little is known about the distribution of *C.carnatus* beyond the sites sampled in this study, multiple anthropogenic activities in the Mukwadzi River catchment raise concerns about the conservation status of this species.

The description of *C.carnatus* contributes towards clarifying the taxonomic uncertainty surrounding species of the genus *Chiloglanis* found within the geographic range formerly attributed to *C.neumanni* within southern Africa. The discovery of *C.carnatus* follows the common pattern found among recent taxonomic studies within the region whereby comprehensive sampling across poorly explored regions and the use of integrated taxonomic approaches has identified unique diversity within species previously thought to have wide distribution ranges (Bragança et al. 2020; Kambikambi et al. 2021; Mazungula and Chakona 2021). This pattern is likely to be consistent across southern Africa suggesting underestimation of the region’s biodiversity. In particular, species such as those from the genus *Chiloglanis* are likely to be more diverse since they occur in disjunct distributions in rheophilic habitats, which are likely to be associated with allopatric speciation. This study also raises the awareness of the potential unique riverine diversity of the rivers that flow through the Great Dyke, an important geological feature where 20 endemic plant species that are adapted to the unique serpentine soils have been recorded (Wild 1965). Further exploration of the aquatic fauna of this poorly surveyed region is likely to uncover additional new species for science.

## Supplementary Material

XML Treatment for Chiloglanis carnatus

## ﻿Additional information

### Author ORCIDs

Tadiwa I. Mutizwa https://orcid.org/0000-0003-4017-1720

Wilbert T. Kadye https://orcid.org/0000-0002-5273-8360

Pedro H. N. Bragança https://orcid.org/0000-0002-8357-7010

Taurai Bere https://orcid.org/0000-0002-8603-5137

Albert Chakona https://orcid.org/0000-0001-6844-7501

### Data availability

All of the data that support the findings of this study are available in the main text.
